# Quantum mechanical lateral force on an atom due to matter wave

**DOI:** 10.1016/j.heliyon.2023.e23449

**Published:** 2023-12-12

**Authors:** Sadia Humaira Salsabil, Golam Dastegir Al-Quaderi, M.R.C. Mahdy

**Affiliations:** a Department of Electrical & Computer Engineering, North South University, Bashundhara, Dhaka 1229, Bangladesh; b Department of Physics, University of Dhaka, Dhaka 1000, Bangladesh

**Keywords:** Quantum simulation, Lateral force, Matter wave manipulation

## Abstract

The area of trapping the atoms or molecules using light has advanced tremendously in the last few decades. In contrast, the idea of controlling (not only trapping) the movement of atomic-sized particles using matter waves is a completely new emerging area of particle manipulation. Though a single previous report has suggested the pulling of atoms based on matter-wave tractor beams, an attempt is yet to be made to produce a lateral force using this technique. This article demonstrates an asymmetric setup that engenders reversible lateral force on an atom due to the interaction energy of the matter wave in the presence of a metal surface. Several full-wave simulations and analytical calculations were performed on a particular set-up of Xenon scatterers placed near a Copper surface, with two counter-propagating plane matter waves of Helium impinging in the direction parallel to the surface. By solving the time-independent Schrödinger equation and using the solution, quantum mechanical stress tensor formalism is applied to compute the force acting on the particle. The simulation results are in excellent agreement with the analytical calculations. The results for the adsorbed scatterer case find this technique to be an efficient cleaning procedure similar to electron-stimulated desorption for futuristic applications.

## 1 Introduction

Optical trapping of micrometer-sized objects has branched out to several methods in the past decades. Laser trapping was popularized by Ashkin in 1970 using two counter-propagating beams [1]. Later in 1986, Ashkin et al. made a revolutionary discovery of trapping using a single beam (optical tweezers) [2]. He also showed the techniques for trapping viruses and living bacteria without harming them [3]. For his contributions, Ashkin was awarded the Nobel Prize in Physics in 2018. It is worth mentioning that laser cooling and trapping have also experienced tremendous advancement in the last few decades. For the development of methods of cooling and trapping atoms with laser light, the 1997 Nobel Prize in Physics was awarded to Claude Cohen-Tannoudji, Steven Chu, and William Daniel Phillips [4].

**Fig. 1:**
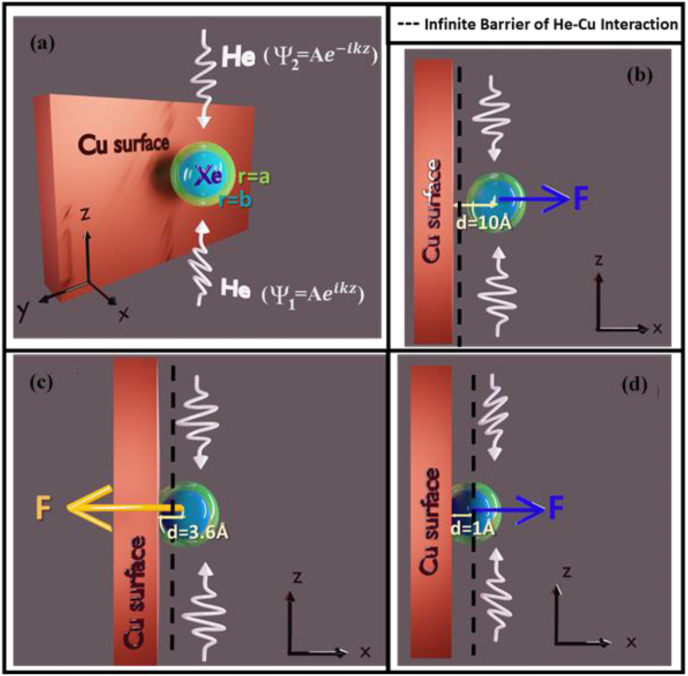
a) The figure shows the geometry of the model in 3D. (b), (c), and (d) illustrate the x−z plane view of the set-up for d=10.0 Å, d=3.6 Å, and d=1.0 Å, respectively, where d is the distance between the scatterer (Xe) and the metal (Cu) surface. Two plane matter waves (He) impinge on the scatterer from the +z and −z directions. The He–Cu physisorption potential energy is infinite on the right-side region of the dashed line. However, the total variation in physisorption potential energy is not shown here. The potential energy for He–Xe is infinite for r<b (blue region), −2.458 meV for b≤r≤a (semi-transparent region), and 0 for r>a, where b=6.697BR and a=9BR, where BR= Bohr radius. (For interpretation of the references to colour in this figure legend, the reader is referred to the Web version of this article.)

Our objective in this work is different from the trapping of atoms as we are focusing on the controlled motion (i.e., manipulation) of quantum objects such as atoms or molecules, etc. In the lateral direction. In addition, instead of applying light beams, this work focuses on manipulation or control of the atoms (i.e., reversible lateral force) by utilizing matter wave beams [cf. Fig. 1 (a)-(d)]. To the best of our knowledge, so far only one article demonstrated a matter-wave-based tractor beam, in which the phenomenon of pulling (instead of lateral force) an atomic-sized scatterer by a Bessel beam matter-wave was utilized [5].

It is well known that light exerts radiation pressure on an object and it pushes a particle towards the direction of its propagation by imparting on the particle momentum carried by the quanta of light, photon. In recent years, macroscopic manipulation via light beams has achieved a completely new level of advancement by using various novel techniques. For example, counterintuitive pulling of dielectric or magneto-dielectric objects using light have been demonstrated in the last decade [[6], [7], [8], [9], [10], [11]]. In addition, the pulling of atoms using electromagnetic waves has also been reported [12,13]. Yet, very few examples include the optical lateral force [[14], [15], [16], [17], [18], [19], [20]], which acts in the direction normal to the direction of incidence of the light wave.

Some particular properties of the incident electromagnetic wave on the scatterer are responsible for the existence of lateral force. The properties of the incident electromagnetic wave can be attributed to its state of polarization or spin-projection in the case of circularly polarized light [14,15]. Other cases of optical lateral force are mostly connected with the chirality of the material [[16], [17], [18]]. However, these attributes are not directly connected with any quantum mechanical set-up or with our work, since in our work, neither the incident particle nor the scatterer possesses any anisotropic property.

Similar to light, quantum mechanical matter wave exerts pressure on an object and it pushes a particle towards the direction of its propagation. This work demonstrates a matter wave-based novel manipulation scenario [cf. Fig. 1 (a)–(d)] that gives rise to reversible lateral force on an atom, due to the interaction of the quantum mechanical matter wave with a metal surface, which creates the required asymmetry. For a symmetric set-up, no lateral force has been observed. We have performed several full wave simulations on a particular set-up of Xenon scatterer atoms placed near the Copper surface, with two plane matter waves of Helium impinging in the direction parallel to the metal surface at a distance from it from the above and below.

In Ref [5], in the set-up of the matter wave tractor beam, the scatterer was modeled by a short-range spherically symmetric potential energy. In our work, we have used Tang-Toennies potential energy [21] to model a realistic scatterer. Despite the spherical potential energy of the target particles in the simulation, the lateral force was achieved by placing the metal surface vertically on one side of the scatterer. The left-right asymmetry was created by the background potential energy resulting from the interaction between the incident particles and the surface (Note that the set-up maintains its symmetry perpendicular to the surface normal). Both analytically and by simulation, there was confirmation of the presence of reversible lateral force.

Adsorption of particles on solid surfaces can be divided into two categories: physisorption, caused by the van der Waals force, and chemisorption, caused by chemical bonds between adsorbate particles and the adsorbent solid [22]. Since one of our objectives is to ensure that there is no chemical bond between the incident particles and the metal, the focus of this paper was on physisorption. We have chosen both the incident particle and the scatterer to be noble gases further ensuring no chemical reaction in the process.

For our proposed set-up, when the target atom was significantly away from the metal, such that their potential energies had zero overlap, it experienced a lateral force away from the metal in the presence of quantum mechanical matter-wave. Due to the minimal overlap scenario, the incident beam was first scattered by the target atom. The initial incident beam did not create any lateral movement of the scatterer because of symmetric scattering in both positive and negative x-directions. However, the portion of the scattered wave traveling towards the direction of metal got reflected back by the infinite barrier of physisorption potential energy. This reflected wave caused a second scattering with the target atom and thus exerted a pushing force on the target atom in the direction away from the metal.

In contrast, when the target atom was at or within the adsorption length from the metal, their potential energies overlapped significantly. The infinite barrier of the physisorption potential covered a significant portion of the scatterer potential energy region (c.f. Fig. 1(b)). As a result, there was significant scattering of the incident particles, from the far side of the scatterer in the direction away from the metal (+x direction). On the near side of the metal, less number of incident particles were scattered from the scatterer resulting in a lesser scattering toward the metal. The total momentum of the scattered wave was, therefore, away from the metal. To conserve momentum, the scatterer gained an equal and opposite momentum towards the metal. However, the scatterer gains energy through the process. If this energy exceeds the energy required for desorption, it should be able to escape the surface. For atomic-sized particles (as incident particles) which share similar adsorption over a metal situation, we adopt a method by utilizing quantum mechanical matter wave (instead of light beams), which may be exploited, depending on the energy of the incident beam and duration of incidence, to extract both chemisorbed and physiosorbed atoms.

In order to use this phenomenon for cleaning the surface, we can keep the substrate vertically or horizontally and in an upside-down orientation. This assures that the impurities do not fall back onto the surface due to gravitational force, which would be the case for a horizontal upside-up surface. A good aspect of this process is that the incident waves do not need to be localized (only their directions have to be maintained), enabling cleaning of a wide surface area.

## 2 Set-up and method

Consider scattering of incident helium atoms by some heavy atom in the presence of external surface energy. Fig. 1 features the schematic diagram of our proposed model, where the heavy atom is situated at a distance d from a semi-infinite surface. The center of the atom is taken as the origin of the coordinate system. We impinge two plane matter waves of the same energy *E* on the scatterer from the +z direction and the −z direction, respectively. Instead of dealing with the entire wave packets which consists of a range of energies, we are considering a plane wave component of energy *E* of the wave packet. Specifically, we have chosen the incident particles to be Helium-4, the scatterer as the atoms of the Xenon gas, and the metal as Copper. Simulation data is generated for d=0 Å to d=10.0 Å (the reason behind starting from d=0 Å is explained in Part 1 of Section A of the supplement material). Furthermore, three specific scenarios have been analyzed for d=10.0 Å (an arbitrarily small distance has been taken ensuring minimum overlap between the He–Cu and He–Xe interaction energies for simplified analysis), d=3.6 Å (adsorption bond length for Xe–Cu taken from Ref. [23]), and d=1.0 Å (the choice of distance is based on the physical explanation, which is discussed in details in the next section).

Unlike in Ref. [5], we cannot use the Center of Mass reference frame, as an external force acts on the particles resulting from the space-varying physisorption potential energy. We will, therefore, use Laboratory reference frame. For the adsorption case, as the target atom vibrates with a small vibration energy [23], it possesses small kinetic energy but no time-averaged momentum. If the vibrational motion is ignored, the target particle can be considered motionless. For simplicity, we are taking the heavy target particles to be motionless for all other *d* as the target particles are around 32 times heavier than the incident particles and are further considered cold. Hence, in the simulation, we solved the Schrödinger equation for the incident particles of free particle mass under the influence of the stationary scatterer and the metal surface.

The data required to model the set-up are, therefore, the free particle mass of the incident beam particles, the energy E of the incident beam, and the total potential energy of system. A constant mass of the incident particle is assumed throughout the entire domain of the system. The beam's energy (25 meV) is given in the same way as in Ref. [5], where a coherent beam with thermal energy at room temperature was provided using an energy selector. The energy is varied from 0 meV to 40 meV for further data analysis. Since the incident particle's energy is a fixed parameter here and the potential energy is time-independent, it was possible to run a stationary study (the supplement includes further information regarding simulation).

**Fig. 2:**
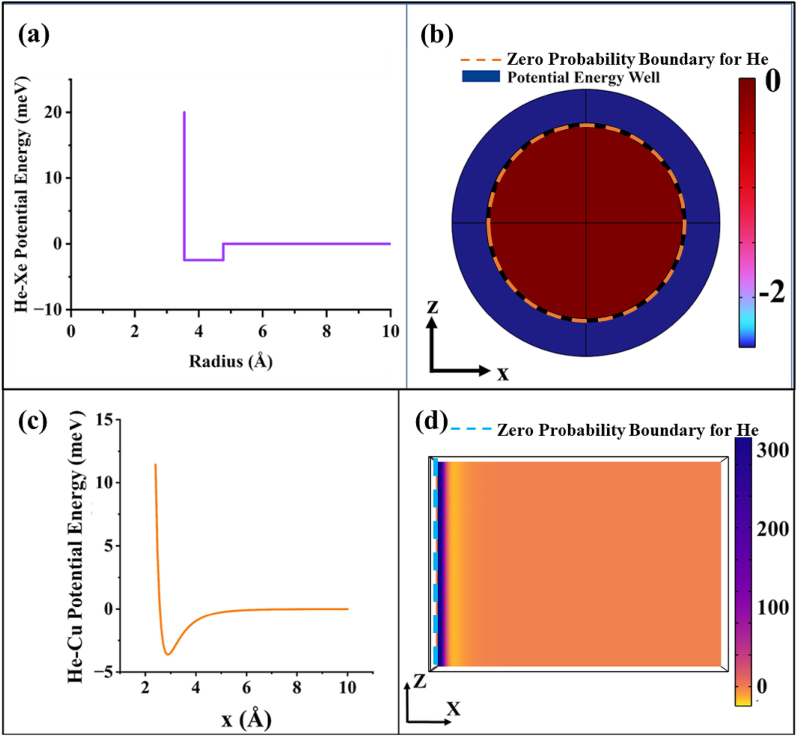
a) The graph represents the step approximation of the potential energy between He and Xe atoms collected from Ref. [21]. b) The figure shows the modeling of potential energy (in meV) between He–Xe in COMSOL. The potential energy is infinite inside the circle representing Zero Probability Boundary. c) This graph represents the experimental potential energy of our incident beam particle (He) in relation to its separation from the Cu surface [25]. Using the information from (c), the potential energy (in meV) of the incident beam under the influence of the metal surface is modeled. d) This figure shows the modeling in COMSOL. The potential energy is infinite on the left region of the straight line representing the Zero Probability Boundary which is at a distance of 2.0 Å from the Cu surface. Figure (c) adapted and redrawn with permission from Ref. 25, © 1977 American Physical Society.

**Fig. 3:**
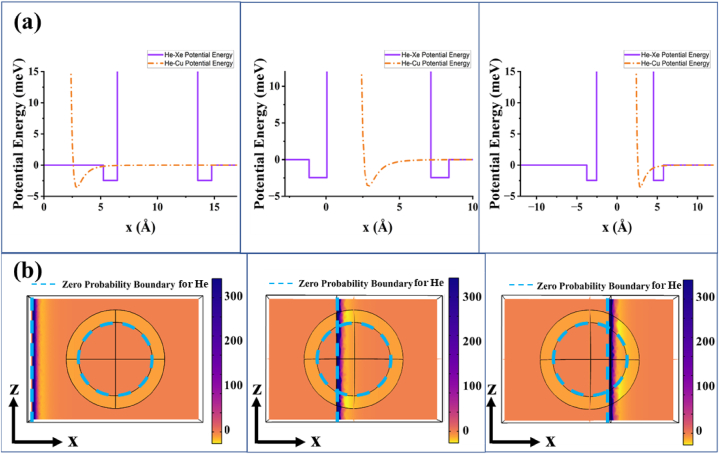
a) The graphs illustrate the potential energy (in meV) overlap shown in Fig. 2, Fig. 3 for d=10.0 Å, d=3.6 Å, and d=1.0 Å, respectively. b) The figures show the x−z view of the net potential energy modeling in COMSOL for d=10.0 Å, d=3.6 Å, and d=1.0 Å, respectively. The potential energy is infinite inside the circle representing Zero Probability Boundary and on the left region of the straight line representing Zero Probability Boundary.

The potential energy of the whole set-up has been divided into two parts: the interaction energy experienced by the incident particles due to the surface (He–Cu potential energy) and that due to the scatterer (He–Xe potential energy). For the first part, although the shape of the interaction energy curve depends on the incident beam energy, Fig. 2(c) was adopted for all the simulations with different incident beam energies. The explanation for this choice is as follows- Given that rare-gas atom is adsorbed over a metallic surface, the shape of the adsorption energy graph would remain similar under different incident beam energy scenarios. Only the repulsive, i.e. the short range, part of the adsorption energy would change due to electronic exchange between the absorbate and metal (as the energized electrons in the adsorbate give rise to different phenomena in the metal, such as photon emission, sputtering, etc. [24]) [25]. The attractive, i.e. the long range, part would remain unaffected (as the adsorbate is far from the surface) [25]. In the Results and Discussion Section, the effect of He–Cu potential energy scaling is demonstrated. No significant changes in the force magnitude were observed and the force trend with respect to distance of Xe from infinite barrier of He–Cu interaction energy also agreed with the prediction made in the next section, validating the use of Fig. 2(c) in the simulations for a wide range of incident beam energies (given that multichannel scattering does not take place, which is out of the scope of this article). For the second part, Tang-Toennies' potential energy is taken into account (the curve and its modeling on COMSOL are shown in Fig. 2(a) and (b), respectively) [21]. The shape of the interaction is considered to be a step approximation of the potential energy curve (this model reduces the complexity of the calculation and allows effective implementation of quantum mechanical (QM) stress tensor [5,26,27] in COMSOL Multiphysics [28]). As the Hamiltonian of a system with time-independent potential energy is the sum of the kinetic energy of the incident particles and the total potential energy, we added both the scalar potential energies as functions of position. The net potential energy model of the set-up is shown in Fig. 3.

The stress tensor has been used to calculate the time-averaged forces acting on a particle. A similar method is used in optics to calculate the time-averaged optical forces using the Minkowski stress tensor [10,29]. As we are considering matter wave as the incident wave, we are using the QM stress tensor instead of the Minkowski stress tensor. The force density vectors in the x, y, and z-directions were calculated and integrated at $r=9BR^{+}$ distance (where BR is the Bohr radius, and the superscript + indicates a slightly larger radius than 9BR, which is 0.1BR for this particular set-up) to produce the respective components of the time-averaged force acting on the particle (further details are given in the supplement). Since we considered the He–Xe potential energy to be negligible or zero for a distance r>9BR, any closed surface at a distance $r>9BR^{+}$ can be used for surface integration.

Using the QM stress tensor derivation for incident particle-scatterer system in Ref. [5], the tensor for our system (incident particle-scatterer-metal) can be derived as follows. Starting with the expectation value expression of force experienced by the incident particle in the total potential energy:

(1) $$F_{inc.}=-\;\int \psi^{*}\;\vec{\nabla }U_{sc.}\;\psi \;dV$$

where, $F_{inc.}$ is the force experienced by the incident particle due to the scatterer, ψ is the total stationary wave function, and $U_{sc.}$ is the potential energy of the incident particle due to the scatterer.

Solving the following TISE of the incident particle in the total potential energy of the system gives ψ:

(2) $$-\;\frac{{\hbar \;}^{2}}{2m}\;\Delta \;\psi +\left(U_{surf.}+U_{sc.}\right)\;\psi \;=E\;\psi$$

where, m is the mass of the incident particles, $U_{surf.}$ is the potential energy of the incident particle due to the surface, and E is the energy of the incident beam.

Applying mathematical identities to the integrand of (1), then applying Eq. (2), and further simplifying gives the following equation:

(3) $$\psi^{*}\vec{\nabla }U\psi =-\vec{\nabla }∙\;[\;\frac{\hbar {\;}^{2}}{2m}\left(\vec{\nabla }\psi ⊗\vec{\nabla }\psi^{*}+\vec{\nabla }\psi^{*}⊗\vec{\nabla }\psi \right)+\overset{═}{I}\;(\left((E-U_{surf.})-U_{sc.}\right){\left|\psi \right|}^{2}+\frac{\hbar {\;}^{2}}{2m}\;{|\vec{\nabla }\psi |\;}^{2})]$$

Therefore,

(4) $$F_{inc.}=\int \vec{\nabla }∙\;[\;\frac{{\hbar \;}^{2}}{2m}\left(\vec{\nabla }\psi ⊗\vec{\nabla }\psi^{*}+\vec{\nabla }\psi^{*}⊗\vec{\nabla }\psi \right)+\overset{═}{I}\;\left(\left((E-U_{surf.})-U_{sc.}\right){\left|\psi \right|}^{2}+\frac{{\hbar \;}^{2}}{2m}\;{|\vec{\nabla }\psi |\;}^{2}\right)]\;dV$$

As the incident particle leaves the scatterer, due to momentum conservation, it exerts an equal and opposite force, $F_{sc.}$, on the scatterer. $F_{sc.}$ can be written in terms of QM stress tensor, $\overset{═}{T}$ , applicable for the scatterer.

(5) $$\left\langle F_{inc.}\;\right\rangle =-\left\langle F_{sc.}\right\rangle =-\left(-\oint \left\langle \overset{═}{T}\right\rangle .d\vec{s}\right)=\oint \langle \overset{═}{T}\rangle \text{.}d\vec{s}$$

where ⟨⟩ denotes time average.

Equating (4) and (5), and applying divergence theorem gives:

(6) $$T=\frac{\hbar^{2}}{2m}\left(\vec{\nabla }\;\psi \;⨂\;\vec{\nabla }\;\psi^{*}+\vec{\nabla }\psi^{*}\;⨂\;\vec{\nabla }\psi \;\right)+\overset{═}{I}\left(\left((E-U_{surf.})-U_{sc.}\right){\left|\psi \right|}^{2}-\frac{\hbar^{2}}{2m}\;{\left|\vec{\nabla }\;\psi \right|}^{2}\right)$$

Using (5), (6), the following formula is obtained:

(7) $${\vec{F}}_{sc.}=-\frac{\hbar^{2}}{2m}\left[r^{2}\oint \left(2\;Re\;\left[\;\vec{\nabla }\;\psi \;\left(\vec{\nabla }\;\psi^{*}\;.\;\hat{n}\right)\right]+\hat{n}\;\left(\frac{2m\left(E-U_{surf.}\right)}{\hbar^{2}}\;{\left|\psi \right|}^{2}-{\left|\vec{\nabla }\;\psi \right|}^{2}\right)\right)\;d\Omega \right]$$

where, $\hat{n}$ is the outward unit vector. From here, it is evident that $\left|{\vec{F}}_{sc.}\right|\propto {\left|A\right|}^{2}$ where A= amplitude of the incident beam.

By applying the QM stress tensor method, the time-averaged pulling force for the set-up of Ref [5] has been verified by comparison with the analytical results in Ref. [5] as a first exercise using full-wave simulation method of COMSOL (detailed results have been given in the supplement). After that, we adopt our set-up which is quite different from the set-up presented in Ref. [5]. A short description of our set-up along with the required boundary conditions applied in the COMSOL software (for the full-wave simulation) is given in the supplement of this article.

## 3 Physical explanation and analytical result

The overall lateral force acting on the scatterer can be explained by three possible scenarios: (A) When there is minimal overlap between the target atom's potential energy and the infinite barrier of the physisorption potential energy; (B) When there is an overlap, but the direction of a significant portion of the incident beam (before interaction with the target atom) remains unaltered by the influence of the physisorption potential energy; and (C) When there is a greater overlap so the direction of a significant portion of the incident beam (before interaction with the target atom) changes by the influence of the physisorption potential energy. Depending on the amount of contribution of each scenario, the lateral force's direction and magnitude change.

The first scenario can be divided into three consecutive events: (i) the single-channel scattering of incident particles by the scatterer (since the incident particles are far away from the incident atom-metal potential energy barrier), (ii) subsequent interaction of the waves scattered in the previous event, with the physisorption potential energy of the incident atom-metal, and (iii) finally, a second single-channel scattering of the resulting reflected wave by the metal with the scatterer Xe atom. Although time-independent scattering theory can be applied in the case of our time-independent potential energy, the limitations of the available analytical method for such asymmetric potential energy led us to divide the events sequentially.

For a simplified calculational procedure, we are considering the value of d equal to 10 Å, such that there is no overlap between the physisorption potential energy of the metal-incident atom and the scatterer-incident atom potential energy. The first event is a 3D scattering of plane waves by a spherical potential energy, allowing us to use the Partial Wave Analysis method for deriving the total wave function. The scattering amplitude is then used to find the portion of the wave scattered toward the metal. Our target is to find non-zero scattering amplitude for θ=π/2, where θ is the polar angle with respect to the incident beam coming from the −z direction in the x-z plane.

The incoming plane waves can be written in terms of spherical waves using Rayleigh's formula as

$$\psi_{inc}\left(r,\theta \right)=A\;e^{ikz}+A\;e^{-ikz}$$

(8) $$=\sum_{l=0}^{\infty }Ai^{l}\;\left(2l+1\right)\;j_{l}\;\left(kr\right)\;P_{l}\left(\cos\;\theta \;\right)+\sum_{l=0}^{\infty }Ai^{l}\;\left(2l+1\right)\;j_{l}\left(-kr\right)\;P_{l}\;\left(\cos\;\theta \right)$$

where A is the amplitude of the incident wave, l is the orbital angular momentum quantum number, $j_{l}$ is the l-th order spherical Bessel function, and $P_{l}$ is the l-th order Legendre polynomial. Since our incident wave function is independent of φ(z=rcosθ), only the m=0 term survives; therefore, we have omitted all the m≠0 terms.

Expanding Eq. (8) in terms of spherical Hankel's function of the first and second kinds, which go to $e^{+ikr}/kr$ and $e^{-ikr}/kr$, respectively for kr≫1, and including phase shift, $\delta_{l}\text{,}$ of the outgoing waves due to the spherical potential energy, we get the overall wave function:

(9) $$\begin{array}{c} \psi \left(r,\theta \right)=A[e^{ikz}+e^{-ikz}\\ +\left[\sum_{l=0}^{\infty }\left(2l+1\right)\frac{1}{k}e^{2i\delta_{l}}\;\sin\;\left(\delta_{l}\right)P_{l}\left(\cos\;\theta \right)+\sum_{l=0}^{\infty }\left(2l+1\right)\frac{1}{k}e^{2i\delta_{l}}\;\sin\;\left(\delta_{l}\right)P_{l}\left(\cos\;\theta \right)\;{\left(-1\right)}^{l}\right]\frac{e^{ikr}}{r}]\; \end{array}$$

where, the first two terms are the incident plane waves functions, and the last term is the scattered spherical wave function. The coefficient of the last term gives the scattering amplitude at different polar angles, θ.

We are considering no interaction between the incident particles by providing a rarefied incident beam. The phase shift is therefore only due to the incident atom-target atom interaction. From Eq. (9), the overall scattering amplitude for the first scattering event can be written as (the derivation is included in the supplement)

(10) $$f_{overall}\left(\theta \right)=\sum_{l=0}^{\infty }\left(2l+1\right)\frac{1}{k}e^{2i\delta_{l}}\;\sin\;\left(\delta_{l}\right)P_{l}\left(\cos\left(\theta \right)\right)\left[1+{\left(-1\right)}^{l}\right]$$

For this, the l-th partial wave amplitude relation with $\delta_{l}$ is given by the following relation:

(11) $$a_{l}=\frac{1}{2ik}\left(e^{2i\delta_{l}}-1\;\right)=\frac{1}{k}e^{2i\delta_{l}}\;\sin\;\left(\delta_{l}\right)$$

To get the scattering amplitude, we will apply the required boundary conditions. The scatterer potential energy is modeled as follows:

$$V\left(r\right)=\left\{ \begin{array}{c} \infty ,\left(r<b\right)\\ -V_{0},\left(b\leq r\leq a\right)\\ \;0,\left(r>a\right) \end{array}\right.$$

where $V_{0}=2.458\;$ meV, a = 9 × Bohr Radius, and b = 6.697 × Bohr Radius for our model. Applying the following three appropriate boundary conditions at r=a and r=b gives Eqs. (12), (13) (detailed calculation is given in the supplement).

(i) $$\psi \left(r=a^{-}\right)=\psi \left(r=a^{+}\right)$$

(ii) $${\frac{\partial \psi }{\partial r}|}_{r=a^{-}}={\frac{\partial \psi }{\partial r}|}_{r=a^{+}}$$

(iii) ψ(r=b)=0

(12) P/Q=R/S

where,

$$P=i^{l}j_{l}\left(ka\right)\left[1+{\left(-1\right)}^{l}\right]+ki^{l+1}a_{l}h_{l}^{\left(1\right)}\left(ka\right)\left[1+{\left(-1\right)}^{l}\right]$$

$$R=i^{l}j_{l}\left(k^{\prime }a\right)\left[1+{\left(-1\right)}^{l}\right]+k^{\prime }i^{l+1}a_{l}^{\prime }h_{l}^{\left(1\right)}\left(k^{\prime }a\right)\left[1+{\left(-1\right)}^{l}\right]$$

$$Q=i^{l}\frac{1}{2}k\left[j_{l-1}\left(ka\right)-j_{l+1}\left(ka\right)\right]\left[1+{\left(-1\right)}^{l}\right]+ki^{l+1}a_{l}\frac{1}{2}k\left[h_{l-1}^{\left(1\right)}\left(ka\right)-h_{l+1}^{\left(1\right)}\left(ka\right)\right]\left[1+{\left(-1\right)}^{l}\right]$$

$$S=i^{l}\frac{1}{2}k^{\prime }\left[j_{l-1}\left(k^{\prime }a\right)-j_{l+1}\left(k^{\prime }a\right)\right]\left[1+{\left(-1\right)}^{l}\right]+k^{\prime }i^{l+1}a_{l}^{\prime }\frac{1}{2}k^{\prime }\;\left[h_{l-1}^{\left(1\right)}\left(k^{\prime }a\right)-h_{l+1}^{\left(1\right)}\left(k^{\prime }a\right)\right]\left[1+{\left(-1\right)}^{l}\right]$$

(13) $$a_{l}^{\prime }=\frac{-[j_{l}\left(k^{\prime }b\right)[1+{\left(-1\right)}^{l}]]}{ik^{\prime }h_{l}^{\left(1\right)}\left(k^{\prime }b\right)\left[1+{\left(-1\right)}^{l}\right]}$$

where k’ = $\sqrt{2m\left(E-\left(-V_{0}\right)\right)}/\hbar$. We used Eqs. (12), (13) to get $a_{l}$, and finally $f_{overall}\left(\theta =\pi /2\right)$ using Wolfram Mathematica. The l-th terms are taken up to ka=33 (where $k=\sqrt{2mE}$/ ħ and E=25 meV; the calculation is shown in the supplement). This is because it is a high-energy scattering for which l≤kr terms should be taken into account (where r is the radius of the spherical potential energy).

The scattering amplitude for θ = π/2 is therefore:

(14) $$f\left(\theta =\frac{\pi }{2}\right)=\sum_{l=0}^{33}[\left(2l+1\right)a_{l}P_{l}\left(0\right)[1+{\left(-1\right)}^{l}]\;$$

Using Eq. (14), we found the magnitude of the scattering amplitude at θ=π/2 for step approximated He–Xe potential energy model and E=25 meV to be $\;4.2204\times 10^{-10}$ $\sqrt{m^{-3}}$ (Mathematica results have been provided in the supplemental material).

The shape of the interaction energy graph for physisorption is an essential part of the second event and can be explained by van der Waals attraction and Pauli repulsion. As the electron wave functions of the adsorbent and adsorbate overlap, the attractive van der Waals force creates potential wells above the surface of a depth of around a few meV. On the other hand, the system's energy increases due to the orthogonality of the wave functions according to the Pauli exclusion principle, which states that no two fermions (in this case, the electron clouds of the particles and the metal atoms) can be in the same quantum state. Pauli exclusion and consequent repulsion are strong for noble gases, dominating the short-range interaction between our incident particles and the metal surface. This combination of short-range Pauli repulsion and long-range van der Waals attraction results in a one-side steep or bounded and one-side open or flat curve of potential energy which allows the scattered wave toward the metal to be reflected back from the bound side and escape through the open side.

For simplicity, only the portion of the scattered plane waves (from the previous event) with k vector in the −x direction is considered, taking into account their maximum contribution to the current event.

$$\psi_{scattered}=\frac{f\left(\theta =\frac{\pi }{2}\right)}{\left|x\right|}Ae^{-ikx}$$

The reflected wave will be in the form:

(15) $$\psi_{reflected}=Be^{ikx}=R_{c}\frac{f\left(\theta =\frac{\pi }{2}\right)}{\left|2d-x-\varepsilon \right|}\;e^{i\delta }Ae^{ikx}$$

where ε = $10^{-12}$ *m* is a small distance (used in order to avoid numerical error), $R_{c}$ is the reflection coefficient for the reflection of He from the He–Cu potential energy barrier, and δ is the phase shift of the reflected wave due to this potential energy. Using 1D simulation in COMSOL, the numerical value of $R_{c}$ is computed to be 0.99927.

$\psi_{reflected}$ is the incident beam for the second scattering event. When incident plane waves impinge on the scatterer from a single direction, only pushing force in the direction of incidence is exerted on the scatterer [5]. Similarly, for this case, the +x direction component of the momentum of the scattered wave from the second scattering event will be less than that of the incident beam ($\psi_{reflected}$), and hence due to momentum conservation, the scatterer gains momentum in the positive +x direction. Therefore, the lateral force on the scatterer will be in the +x direction. Using the analytical values of $R_{c}$ and $f\left(\theta =\frac{\pi }{2}\right)\text{,}$ the analytical magnitude of the lateral force for E = 25 meV and *d* = 10 Å should be ${\left(R_{c}\left|\frac{f\left(\theta =\frac{\pi }{2}\right)}{2d-a}e^{i\delta }\right|\right)}^{2}F_{z}\approx$ $0.077F_{z}$ = $1.46\times 10^{-20}$ N, where $F_{z}$ is the pushing force exerted by a single plane matter-wave (cf. Table 1).

**Table 1:** This table represents the force components acting on the scatterer in the absence of the metal surface for E=25 meV. From here, we can conclude that $F_{x}$, $F_{y}$, and $F_{z}$ can be considered zero for the incidence of plane waves from the +z and −z directions set-up, because these values are insignificant compared to $F_{z}$ for the set-up where the incidence of plane waves is in the +z direction (any value in the scale of $10^{-21}$ or less is considered negligible or zero). The non-zero values of $\left\langle F_{x}\right\rangle$, $\left\langle F_{y}\right\rangle$, and $\left\langle F_{z}\right\rangle$ for the two-beam scenario are due to small computational error.

Incident Beam Direction	$F_{x}$ (N)	$F_{y}$ (N)	$F_{z}$ (N)
−z**and**z	3.29 $\times 10^{-22}\approx 0$	−1.09 $\times 10^{-22}\approx 0$	6.93 $\times 10^{-23}\approx 0$
z	3.72 $\times 10^{-23}\approx 0$	−3.97 $\times 10^{-23}\approx 0$	$1.89\times 10^{-19}$

For the second scenario, since the potential energy modeling is NOT spherically symmetric, the analytical solution of the time-independent Schrödinger equation (TISE) is intractable. The scattered particles leave the potential energy as a plane wave regardless of the shape of the potential energy. The direction of the net force on the scatterer can be obtained due to significant rightward scattering (in the +x direction) compared to insignificant leftward scattering (as the potential barrier blocks the majority of the left side volume of the scatterer's potential energy, therefore blocking the scattered wave from leaving through the left side). Since the final total momentum of the scattered wave is in the +x direction, the scatterer gains equal and opposite momentum to conserve the total zero lateral momentum of the system. The lateral force on the scatterer will, therefore, be in the −x direction.

For the third scenario, the analytical solution of TISE is also intractable. In this case, a significant portion of the incident beam goes through a change of direction before getting scattered by the target atom. As the x-axis gradient of He–Cu physisorption potential energy is higher on the left side of the He–Cu potential energy minima (steeper slope on the left side) than to the right (cf. Fig. 2(c)), the incident beam changes direction more to the right due to gradient force ($\vec{F}=-\vec{\nabla }\;U$). Therefore, it exerts a lateral force to the right (+x direction).

## 4 Results and discussion

**Fig. 4:**
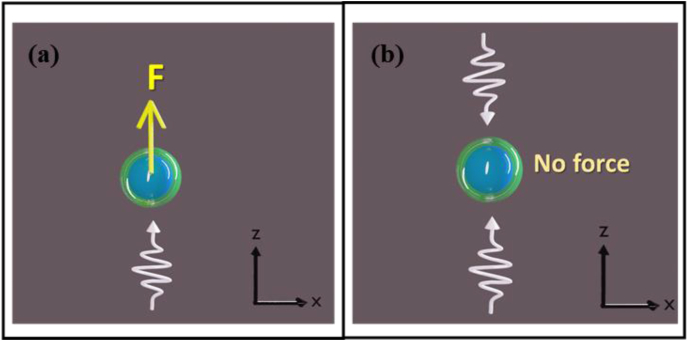
Figures (a) and (b) show the set-up for the counter-propagating incident beam and single incident beam, respectively, in the absence of the Copper surface. In both cases, there was no lateral force experienced by the scatterer.

We verified that for symmetric set-up (in the absence of the Copper surface), there is no lateral force. The set-up is shown in Fig. 4, and the results are shown in Table 1 for E=25 meV.

**Fig. 5:**
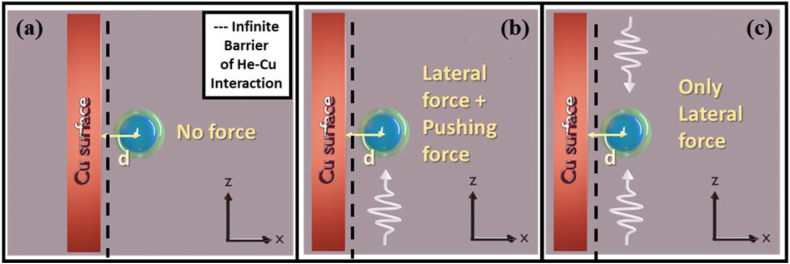
Figures (a), (b), and (c) illustrate the x−z plane view of the setup for no incident beam, single incident beam, and counter-propagating incident beams, respectively, where d is the distance between the scatterer (Xe) and the metal (Cu) surface. (a) No force was observed for the no incident beam scenario b) Lateral force, as well as pushing force in the +z direction was observed for a single incident beam. (c) Only lateral force was observed in this case, as the vertical forces got canceled out. To ensure only lateral force, we chose this as our main setup.

**Fig. 6:**
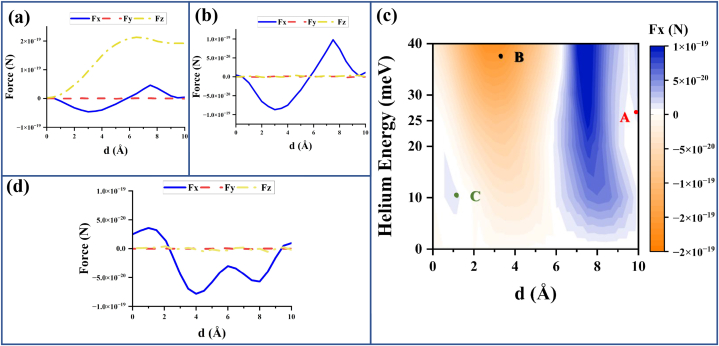
Subfigures (a), (b), and (c) are the simulation results using step-approximated He–Xe interaction energy, while (d) is the simulation result using the actual He–Xe interaction energy curve. a) This graph represents the force components on the scatterer for variation in distance from the scatterer to the metal (i.e., d) and the incidence of plane matter waves in the +z direction only (from a single direction). From here, we can conclude that there is a net lateral force on the scatterer in the −x direction for small *d* and in the +x direction for large *d*. Moreover, a pushing force in the +z direction is also observed. b) This graph represents the force components on the scatterer for variation in d and the incidence of plane matter waves from +z and −z directions (counter-propagating plane matter waves). From here, we can conclude that there is a net lateral force (the most dominant component of force among all the components) acting on the scatterer in the −x direction for small *d* and in the +x direction for large *d*. c) The graph represents the variation in $F_{x}$ (lateral force) for different values of d and E (energy of the incident particles). Points A, B, and C represent the setups where the first, second, and third scenarios dominate, respectively. d) Lateral force (blue) is observed to be following the same trend (from +x to −x to +x direction) as expected. Between *d* = 2 Å to 2.5 Å, the physisorption potential energy infinite barrier crosses the center of the atom, therefore, the first transition is observed here. The infinite barrier meets the scatterer's potential energy between *d* = 9.5 Å to 10 Å where the second transition occurs. $F_{y}$ and $F_{z}$ are negligible as expected. (For interpretation of the references to colour in this figure legend, the reader is referred to the Web version of this article.)

The set-up for simulation in the presence of a Copper surface and no incident beam is shown in Fig. 5(a), where the scatterer experiences no force. In Fig. 5(b), a single incident beam is impinged on the scatterer in the +z direction, and the results for E=25 meV are given in Fig. 6(a). From the figure, it can be observed that the scatterer experiences a significant lateral force in the presence of the metal surface while a pushing force is also present.

Since we are looking for a lateral force, we introduce a second beam incident in the −z direction resulting in a pair of counter-propagating incident beams as shown in Fig. 5(c). The results for simulation in the presence of copper surface and counter-propagating incident beams are given in Fig. 6(b) for E=25 meV. There is no pushing force as seen in the figure. In the previous section, we saw that the magnitude of lateral force for d=10.0 Å should be $1.46\times 10^{-20}$ N, which is very close to the lateral force obtained from the simulation ($1.11\times 10^{-20}$ N). We can say that the difference has occurred due to the assumptions made regarding the reflected wave. The force variation due to change in d for different incident energies *E* is illustrated in Fig. 6(c). Two specific patterns of the lateral force direction with respect to decreasing distance from the surface can be inferred from the figure. The pattern for E = 10 meV changes from +x direction to −x direction and again back to +x direction, whereas the pattern for higher energies goes from +x direction to −x direction and does not reverse back to +x direction. Point A (E = 25 meV and *d* = 10 Å) represents the force from the first scenario, therefore, the lateral force is in the +x direction. Similarly, Point B represents the force where the second scenario dominates, therefore, the lateral force is in the −x direction. As discussed in the third scenario, it can be seen that at point C (*E* = 10 meV and *d* = 1.0 Å), the lateral force is in the +x direction. Table 2 represents the simulation result for force components for Xe–Cu adsorption bond length distance.

**Table 2:** This table represents the force components for the scatterer distance of 3.6 Å from the metal and the incidence of plane waves from both the z and −z directions. From here, we can conclude that a lateral force in the −x direction is experienced by the scatterer.

Distance from the metal surface	$F_{x}$ (N)	$F_{y}$ (N)	$F_{z}$ (N)
**d=3**.**6** **Å**	$-1\times 10^{-19}$	$-1\times 10^{-22}\approx 0$	$-1\times 10^{-21}\approx 0$

For the adsorption case, the lateral force acts towards the surface (in −x direction). As far as our concern is, this force causes the scatterer wave packet to move toward the surface by gaining a wave vector component in the −x direction. However, due to momentum transfer, the kinetic energy rises, and therefore, the thermal energy of the scatterer rises (initially the scatterer's energy is negative of binding energy due to the exothermic process of adsorption [30]). Given that the variation of the Xe–Cu potential energy (see Ref. [23]) with respect to the *d* is similar to that of He–Cu (shown in Fig. 2(c)), when the scatterer's thermal energy exceeds the adsorption binding energy, it will be desorbed through the open side of the curve. Moreover, to further ensure cleaning, vacuum pumping can be introduced.

The initial total momentum of the incident beams is zero as the beams are counter-propagating. The transferred momentum from the incident particle to the scatterer would be maximum if the model is such that the magnitude of the scattering amplitudes divided by a for either θ=π/2 or θ=−π/2 are 1 (i.e., the maximum value, as it is a probability amplitude) for each of the incident beams.

Given a scenario where two counter-propagating incident atoms are scattered by a scatterer atom, the total transferred momentum $p_{sc.}$ can, therefore, vary from 0 to $p_{{sc.}_{\max}}=2p_{inc.}$ where 2 is the total scattering amplitude contributing to the lateral force, and $p_{inc.}$ is the momentum of each incident beam. Applying *E* = $\frac{p^{2}}{2m}$, the following relation between incident beam energy ($E_{inc.}$) and maximum energy transferred to the scatterer ($E_{{sc.}_{\max}}$) can be found:

$$E_{{sc.}_{\max}}=\frac{4m_{inc.}}{m_{sc.}}E_{inc.}$$

where, $m_{inc.}$ and $m_{sc.}$ are the mass of incident atom and scatterer atom, respectively. The factor 4 is resultant from $p_{{sc.}_{\max}}=2p_{inc.}$ relation. Applying this scenario to Helium incident particle and Xenon scatterer at room temperature would give the following $E_{{sc.}_{\max}}$.

(16) $$E_{{sc.}_{\max}}=\frac{4m_{He}}{m_{Xe}}\;E=\left(\frac{4\times 4}{131}\times 25\right)\;meV$$

From the above relation, we get the maximum energy transferred for each scattering event of our set-up is around 3 meV. However, if the incident particle energy is high (for example, around 2 eV), $E_{\max}$ would be around 244 meV, which exceeds the adsorption binding energy of Xe–Cu (111) (173–200 meV) [23]. Hence, depending on the incident particle energy and the scattering amplitude, it is possible to overcome the adsorption binding energy. Moreover, no Helium would be adsorbed as its binding energy is very small (4 meV) compared to its incident energy (25 meV). The shape of the incident particle-metal physisorption energy makes such an incident particle suitable for cleaning as the incident particles are unable to tunnel to the surface.

A real-world example where this technique can be utilized is to clean graphene surfaces. The interaction of some atomic-sized particles with the graphene surface also has similar adsorption energy curves as He-Cu [31]. Hence, such atomic-sized particles can be used as incident particles to remove unwanted particles from graphene before using it as a semiconductor for modern electronics [32].

Fig. 6(d) shows the simulation result using the actual potential energy curve of the He–Xe interaction. The surface integration was taken over a Gaussian surface of radius of 15BR considering the potential energy becomes negligible at this radius. Although the force direction for *d* = 3.6 Å and 1 Å is consistent with that for the setup using step-approximated He–Xe potential energy, the distance at which the transition of the direction from −x direction to +x direction occurs should be different as the radius at which the potential energy becomes zero/negligible is different for each setup. Apart from this difference, the step-approximation accommodated well as an alternative to the phase shift by the actual He–Xe potential energy. Therefore, any scenario can be observed by scaling the parameters, such as potential energy well scaling, radial scaling, etc.

**Fig. 7:**
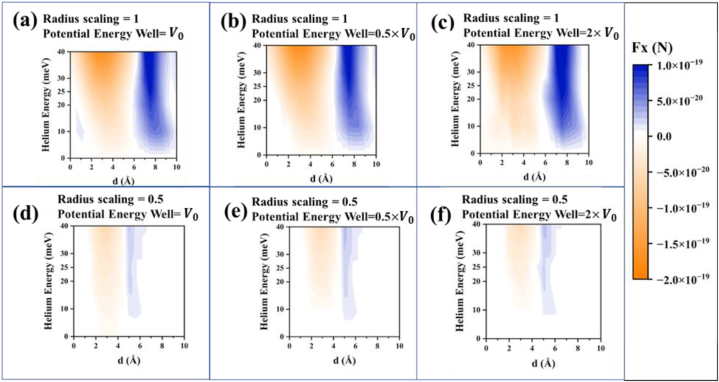
Lateral Force in the +x direction (blue) and in the −x direction (orange) for step-approximated target atom-scatterer potential energy. The dependence of force on the potential well and radial scaling is presented here. (a) Shows the result for the shape of He–Xe Potential energy shown in Fig. 2, (b) shows the result when the potential energy well $V_{0}$ is scaled to half, and (c) when $V_{0}$ is scaled to double, where $V_{0}$ = 2.458 meV. The physisorption potential barrier, in this case, meets the scatterer's potential energy when *d* = 6.76 Å. The transition from +x direction force to −x direction force, as predicted, lies near this distance. (d), (e) and (f) present the result for radial scaling to half. Potential energy well scaling is one, half, and double for (d), (e), and (f), respectively. For radial scaling to half, the physisorption potential barrier meets the scatterer's potential energy when *d* = 4.38 Å and the transition from +x direction force to −x direction force occurs near the meeting point for this case as well. (For interpretation of the references to colour in this figure legend, the reader is referred to the Web version of this article.)

The potential energy curves of different incident atom-scatterer pairs have the same shape but equilibrium length and well depth, and hence, different parameters. Specifically, to observe the variation in lateral force due to the different equilibrium length, radial scaling was carried out. Here, the transition radii of He–Xe potential energy i.e., the spheres in the simulation geometry were scaled (further information on the simulations is provided in the supplement). Moreover, the effect of different well depths, which contributes to different phase shifts, on the force were also observed. The results of these two analyses are presented in each sub-figures of Fig. 7. Fig. 7(a) is the same as Fig. 6(c), and has been included to compare with the other sub-figures (Fig. 7(b–f)). For all the energies, except E = 10 meV in Fig. 7(a), the force is in the −x direction for shorter distances, and the force is in the +x direction for larger distances. From the graphs, we can further conclude that for a greater radial scale, the force magnitude is higher for the same incident particle energies. To get the same force magnitude for a smaller radial scale, the incident beam energy should be increased by the same factor by which the radial scale is decreased. This is because the energy scale is inversely proportional to R [5] (where R is proportional to our radial scaling factor). Lastly, there has not been much of a difference observed due to the potential energy well depth variation. These analyses were carried out to determine the effect of using different incident atom-scatterer pairs.

**Fig. 8:**
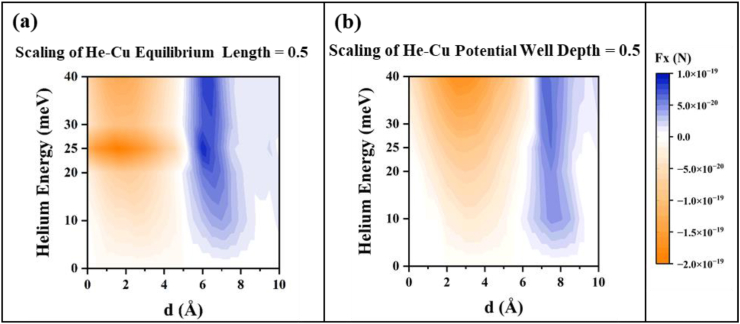
(a) and (b) represent the effect of scaling of the interaction energy between He and Cu surface. Comparing the force magnitudes to Fig. 7(a), no major changes are observed. However, in (a), the force trend is shifted according to the distance between Xe and the infinite barrier of He–Cu interaction energy. In both cases, the third scenario (force in the +x direction for small *d*) is not observed.

Two more analyses (shown in Fig. 8) have been conducted by scaling He–Cu interaction energy parameters, in an attempt to observe their effect on the force scale and pattern with respect to *d*. As mentioned in the Set-up and Method Section, the energy of the incident beam plays a role in altering the parameters of the incident atom-metal interaction energy. In the subfigures, Fig. 8(a–b), it can be seen that the shape of the incident atom-metal interaction energy curve has minimal effect on the force magnitude. On the other hand, from Fig. 8(a), it can be observed Equilibrium Length scaling shifts transition distances in the trend of force direction. This is because, when the equilibrium length is scaled the infinite barrier of incident atom-metal interaction energy is shifted accordingly. To conclude, the results should be similar for a wide range of incident beam energies, which validates our simulations using the same He–Cu interaction energy for different incident beam energies.

## 5 Conclusion

In conclusion, we have presented a simple configuration in which atomic-sized particles can experience a reversible lateral force in the presence of quantum mechanical matter waves resulting from the presence of surface energy. The configuration has been set to reflect a real-world situation and has been verified using full-wave simulation. We have observed three different phenomena: each occurring between two transitions (when the incident atom physisorption energy infinite barrier touches the scatterer-incident atom potential energy, and around a distance when the infinite barrier crosses its center). When the target atom was far from the surface such that their interaction energies with the incident atom did not coincide, two scattering events occurred: first scattering of the incident beam by the scatterer, and second scattering of a portion of the scattered waves (reflected by the physisorption energy infinite barrier) by the scatterer. The second scattering event caused the scatterer to be pushed away from the metal. On the other hand, when the infinite barrier obscured the majority of half of the scatterer's influence region, the incident particles got scattered more in the direction away from the metal than toward it after hitting the scatterer. Therefore, due to momentum conservation, the scatterer was pulled toward the metal. Lastly, when the infinite barrier covered more than half of the scatterer's influence region, a significant amount of incident beam bent away from the metal before hitting the scatterer due to the gradient force by physisorption potential energy. This caused the incident beam to push the scatterer to the lateral direction of incidence of the counter-propagating waves i.e., away from the metal. This manipulation can be utilized in fields where any kind of background potential energy is involved. Additionally, desorption can be possible by using an incident particle that shares similar potential energy with the surface as He–Cu.

## Ethics declaration

Review and/or approval by an ethics committee was not needed for this study because it is based on simulation and analytical studies. There was no involvement of any ethical issues.

Informed consent was not required for this study because no survey and experiment were carried out.

## Data availability statement

Data included in article/supp. Material/referenced in article.

## CRediT authorship contribution statement

**Sadia Humaira Salsabil:** Writing – original draft, Methodology, Investigation, Data curation, Conceptualization. **Golam Dastegir Al-Quaderi:** Writing – review & editing, Validation, Supervision, Conceptualization. **M.R.C. Mahdy:** Writing – review & editing, Validation, Supervision, Resources, Conceptualization.

## Declaration of competing interest

The authors declare that they have no known competing financial interests or personal relationships that could have appeared to influence the work reported in this paper.
