# Supplementary material for: Quantum mechanical lateral force on an atom due to matter wave
Source: Heliyon. 2023 Dec 12;10(1):e23449. doi: 10.1016/j.heliyon.2023.e23449 (PMC10772092; doi:10.1016/j.heliyon.2023.e23449)
Supplement: Multimedia component 1 [file mmc1.docx]

**Supplementary Information for “Quantum Mechanical Lateral Force on an Atom due to Matter Wave”**

Sadia Humaira Salsabil^1^, Golam Dastegir Al-Quaderi^2^, M.R.C. Mahdy^1*^

*^1^Department of Electrical & Computer Engineering, North South University, Bashundhara, Dhaka 1229, Bangladesh*

*^2^Department of Physics, University of Dhaka, Dhaka 1000, Bangladesh*

^*^Corresponding author’s email address: [mahdy.chowdhury@northsouth.edu](mailto:mahdy.chowdhury@northsouth.edu)

**Contents**

| **Introduction………………………………………………………………………** | **3** |
| --- | --- |
| **Section A: Simulation…………………………………………………………….**  SA1: Model Definition………………………………………………………..…………………...  SA2: Results and Discussion………………………………………………………………………  SA3: 3D Simulation of our Set-up for Step Approximated He-Xe Potential Energy……………..    SA4: Verifying the Simulation Method using Matter wave Tractor Beam…………..…………... | **4**  4  5  9  11 |
| **Section B:**  **Mathematical Analysis……………………………………………...** | **13** |
| **Section C: Numerical Solution…………………………………………………..**  SC1: Numerical Solution using Wolfram Mathematica of the Scattering Amplitude for the First Scenario…………………………………………………………………………………………...  SC2: Numerical Solution using COMSOL Multiphysics of the 1D Reflection of the scattered wave from the First Event of the First Scenario…………………………………………………..  SC3: Analysis of the Numerical Data for the First Scenario……………………………………...  SC4: Formulae of He-Cu Potential Energy and He-Xe Potential Energy obtained from Wolfram Mathematica………………………………………………………………………………………. | **18**  18  18  20  20 |

# **Introduction**

This supplement consists of three sections, the first of which introduces the reader to the steps involved in performing the quantum mechanical simulations of the main article, further extending to the simulation of the Matter wave Tractor Beam set-up in Ref. [1]. The second section provides the reader with a detailed procedure of the mathematical analysis discussed in the main article. Finally, the third section provides the reader with further details of the numerical analysis of the first scenario of physical explanation in the main article, along with the process of obtaining the potential energy formulae from the graphs using Wolfram Mathematica.

# **Section A**

## **SA1: Model Definition**

For our set-up, the target atom is modeled using two surface spheres of radius $6.697\times Bohr Radius$(sphere 1) and 9$\times Bohr Radius$(sphere 2), defining the radii with vertical tangents in the step approximated incident particle(He)-target particle(Xe) potential energy graph (Fig. 3 of the main article). Another surface sphere (sphere 3) of radius 9$\times Bohr Radius$ + 0.1$\times Bohr Radius$ is modeled to represent the Gaussian surface over which the QM tensor will be applied. For the Matter wave Tractor Beam set-up, the target atom is modeled using a surface sphere of radius $Bohr Radius$ (sphere 4). Another two surface spheres(sphere 5 and sphere 6) of radius $Bohr Radius$ + 0.1$\times Bohr Radius$and $Bohr Radius$ + 0.3$\times Bohr Radius$, respectively, are modeled to represent the Gaussian surfaces over which the QM tensor will be applied.

For both set-ups, the modeling domain has been created depending on the size of the target atom-incident atom potential energy. The modeling domain for the Lateral Force set-up consists of two solid blocks of a height and depth of 11.64 Å and a total width of around 16.2 Å. The target atom is positioned at the center of the coordinate system. Depending on the arbitrary distance between the center of the atom and the metal surface (i.e., d), the width of each rectangle is varied. The modeling domain for the Matter wave Tractor Beam set-up consists of two solid blocks of a height and depth of $4\times Bohr Radius$ and a width of around $2\times Bohr Radius$ each.


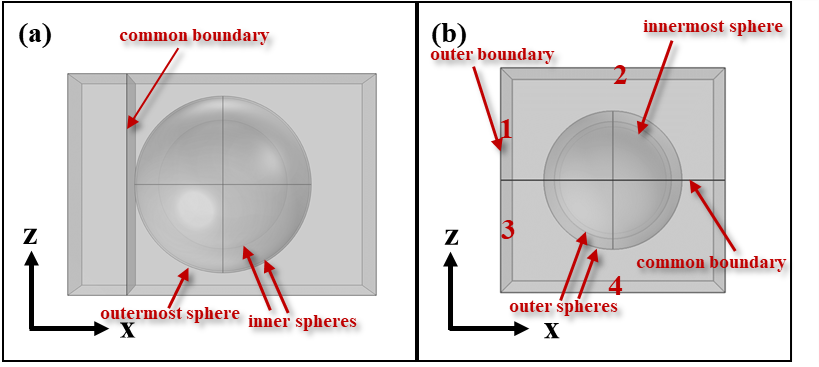


Figure 1S: The Sub-figures (a) and (b) represent the geometries for our set-up using Step Approximated He-Xe Potential Energy, and for the set-up of Matter wave Tractor Beam using plane waves, respectively. For both the set-ups, two solid Blocks are used to design the modeling domains. In (a), the purpose of the two blocks is to apply Zero Probability Boundary of the physisorption energy curve at the common boundary of the blocks, whereas in (b), the purpose is to separate one of the modeling domain’s outer boundaries (into 1 and 3, as labelled in the figure) for the incoming plane waves to enter from two directions (one plane wave enters from 1 and 2, and the other from 3 and 4). a) The two inner surface spheres are the boundaries where the two transitions occur in the He-Xe step-approximated potential energy, and the outmost surface sphere is the gaussian surface where the QM stress tensor is applied. b) The innermost surface sphere is the boundary where the transition from potential energy well and zero potential energy occurs in the Matter wave tractor beam system, and the two outer surface spheres are the gaussian surfaces where the QM stress tensor is applied.

The terminology “Zero Probability Barrier” is according to COMSOL simulation, where the Zero Probability boundary condition is applied on a surface. For a given momentum of matter wave (Helium), 1D simulation has been run to find the ideal distances for He-Cu and He-Xe Zero Probability Barrier (defined by circle and straight line, respectively, in Fig. 3(b) of the manuscript) to ensure that the matter wave attenuates completely before reaching the Zero Probability Barrier. It can be observed using the 1D simulation model in part 2 of Section C (Section SC2) by applying an incoming plane wave of Helium from the direction perpendicular to the potential energy variation of He-Xe and He-Cu in separate simulations. For higher energies of Helium, and hence higher momentum, the complete attenuation distance will be greater. Since 40 meV was the highest energy of Helium in the simulations of our set-up, the Zero Probability Barrier distance was chosen such that the matter wave with 40 meV gets attenuated completely before reaching it.

Similarly, it is true that Xenon cannot cross the Zero Probability Barrier of Xe-Cu potential energy. However, if we consider the restriction, a similar process of finding the Zero Probability Barrier for a given momentum of Xenon atoms towards the surface would have to be applied. Given the fact that there is no limit to the momentum gain, we do not know for certain how close the Xenon atoms can get to the Copper surface before attenuating to zero. Therefore, we have considered all the possible positions starting from $d=0$ Å for the Xenon atoms arbitrarily

COMSOL Multiphysics 5.3a, 5.6, and 6.0 have been used for the COMSOL simulations. Ref. [2] has been used as a reference to carry out the simulations.

## **SA2: Results and Discussion**

For our set-up:

***Observation:***

Fig. 6, 7 and 8 of the manuscript were obtained from the simulations. For every simulation in Fig. 7 of the manuscript with an incident beam energy of 25 meV, the force components in symmetric planes are in the range of ${10}^{-21}$N or less. Whereas the maximum force obtained in the asymmetric plane is on the scale of ${10}^{-19}$N. This exposes our setup to a 1% numerical error or less. The same percentage of numerical error applies to all the other simulations.

For the Matter wave Tractor Beam set-up, five different types of verification have been conducted:

1. Variation of cone angle from 0 to 90° for the set-up described in Section 17 of the supplement of Ref. 1.
2. Surface Integration of the Stress Tensor over two different closed surfaces larger than the radial influence of the incident particle-target atom potential energy in the setup in I.
3. Radial Scaling of the incident particle-target atom potential energy of the setup in I.
4. Comparison of the axial force magnitude and direction for Morse Potential given in Fig. 3(b) of Ref. 1 with our simulated result.
5. Comparison of the axial force magnitude and direction for Coulomb Potential given in Fig. 3(b) of Ref. 1 with our simulated result.
6. Simulation of the setup in I using Bessel Beam.

***Observation:***

Figures 2S, 3S, 4S, 5S, and 6S represent the analysis in I, II, III, IV, and V, respectively. Table 1S represents the analysis in VI.


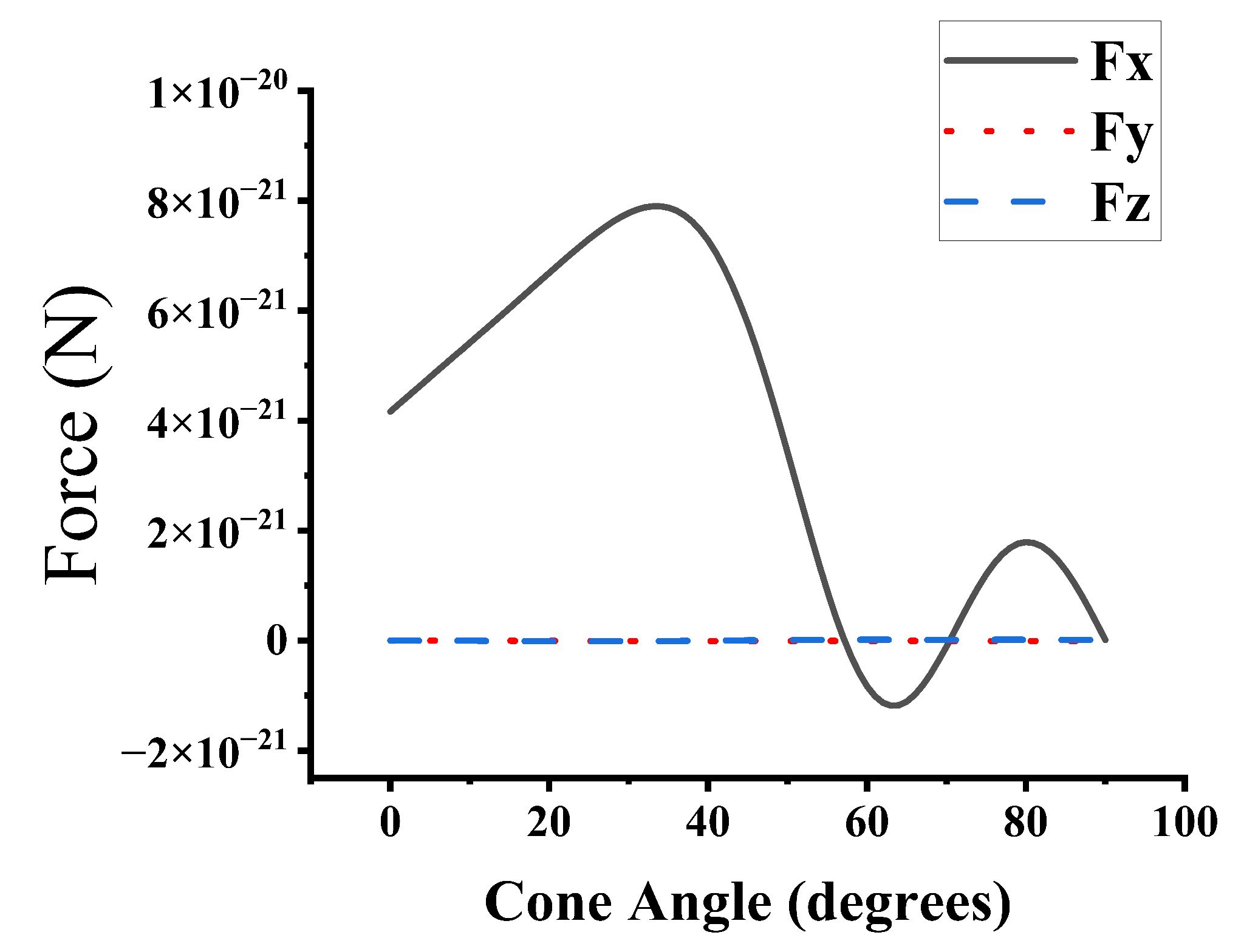


Figure 2S: The graph shows the force components acting on the scatterer with respect to the change in cone angle. As expected, for cone angle = 0°, the particle experiences a pushing force, and for cone angle = 70°, the particle experiences a pulling force at a scale of ${10}^{-22}$ N that agrees with the analytical value obtained in the supplement material of Matterwave Tractor Beam article[1]. The force components in the $F_{y}$ and $F_{z}$ directions are zero (any value less than ${10}^{-23}$ N is considered zero/negligible for this set-up, given that the highest force is in the scale of ${10}^{-21}$ N).


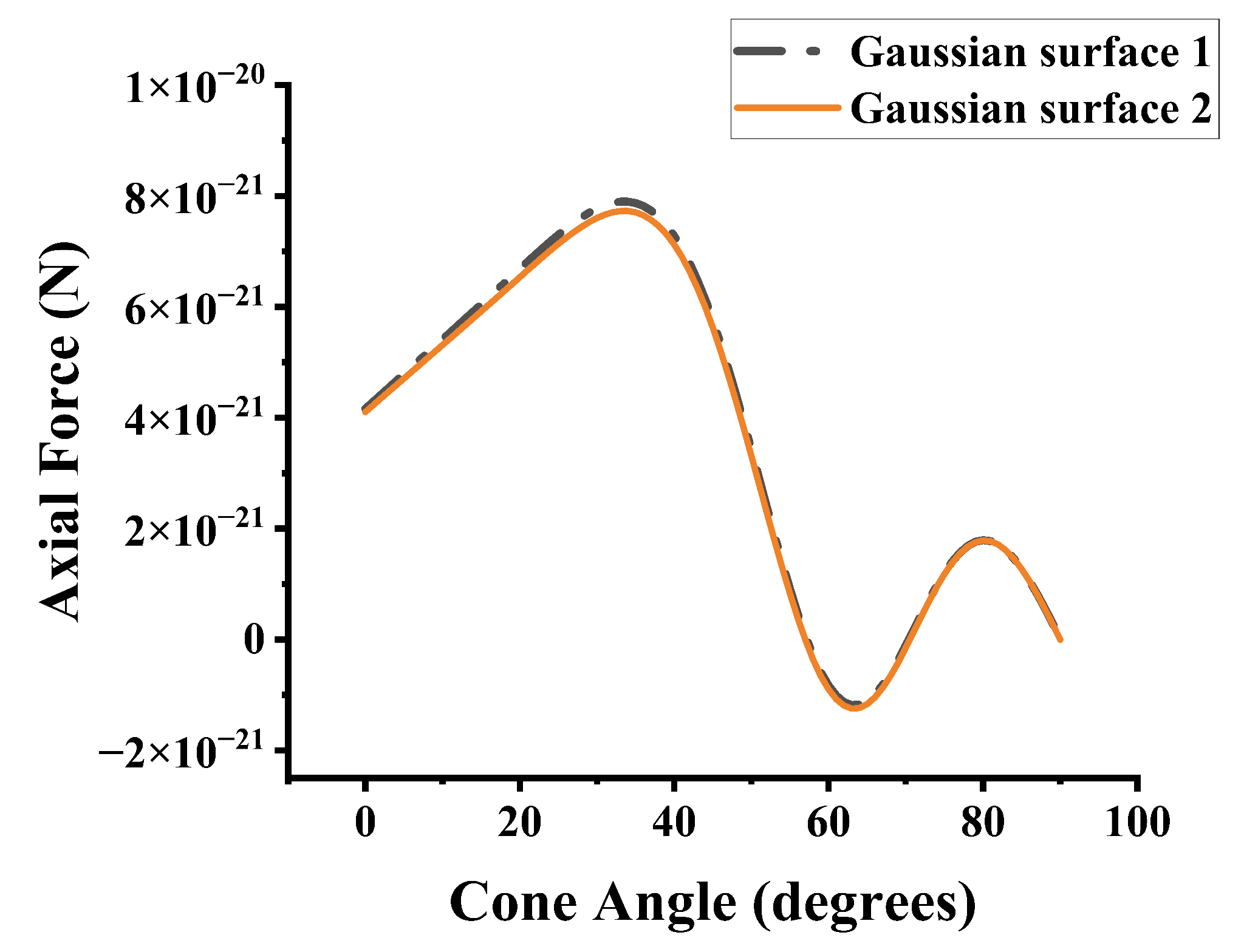


Figure 3S: The graph shows the trend in Axial Force ($F_{x}$) with respect to the change in cone angle for Gaussian surface 1(sphere 5) and Gaussian surface 2(sphere 6). The force magnitude and direction obtained from each Gaussian surface are very close. The model, therefore, satisfies the property of the Stress Tensor, which implies that the force would remain the same upon taking integration over any closed surface of radius greater than the influence of the scatterer (i.e. where incident particle-scatterer potential energy becomes negligible/zero).

For the third analysis, radial scaling is performed, and adjustment of the incident beam energy and the incident atom-target atom potential energy are also carried out, accordingly, to keep the magnitude of $F_{N}$(force scale) the same.


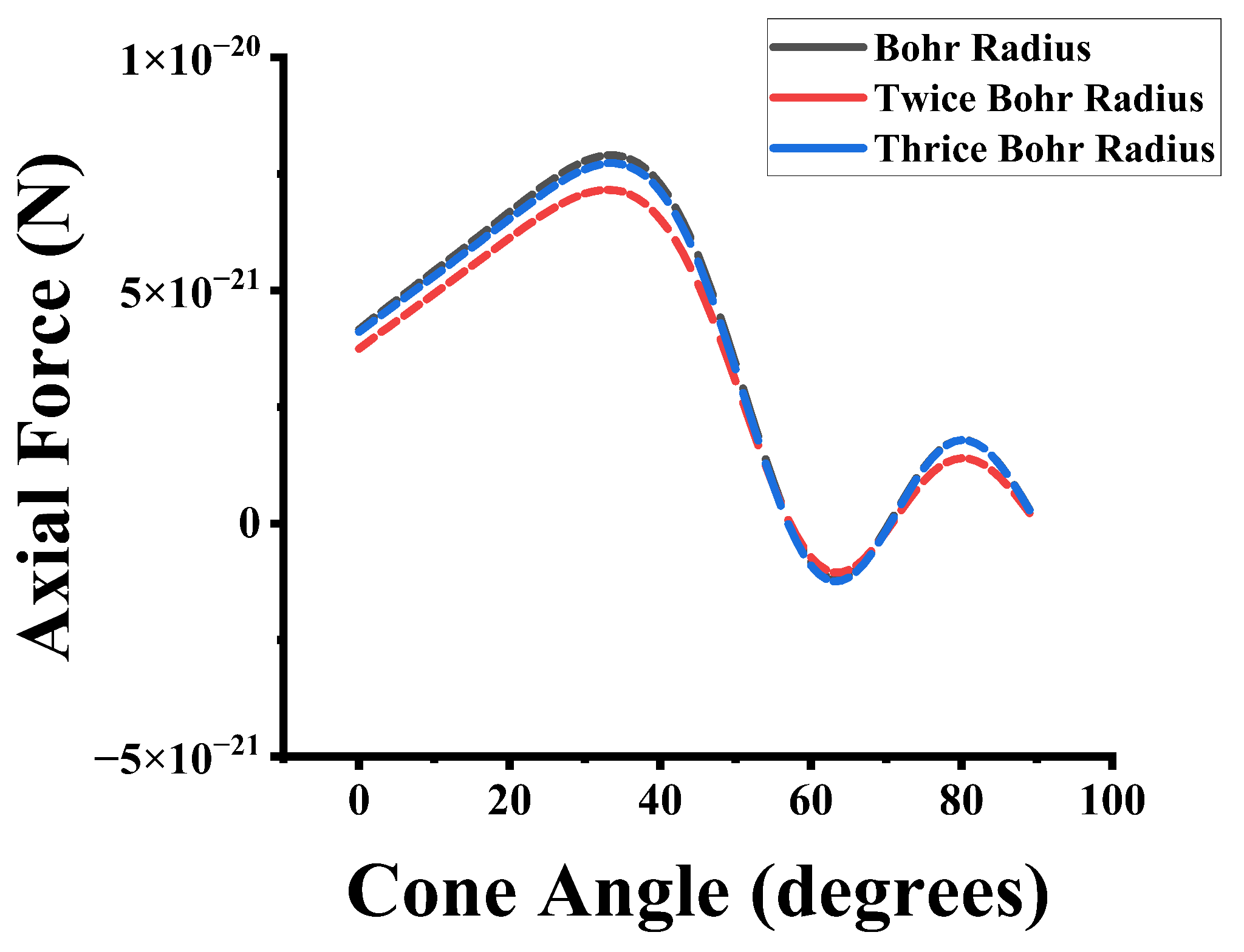


Figure 4S: Black, Red, and Blue curves represent the axial force for the spherical potential energy of a radius $R=Bohr Radius$, R=$2\times Bohr Radius$, and R=$3\times Bohr Radius$, respectively. The respective force values at cone angle= 70 are $-1.05\times{10}^{-22}$ N, $-1.76\times{10}^{-22}$, and$-1.47\times{10}^{-22}$ N. The force magnitudes are very close, satisfying the independence of radial scaling.


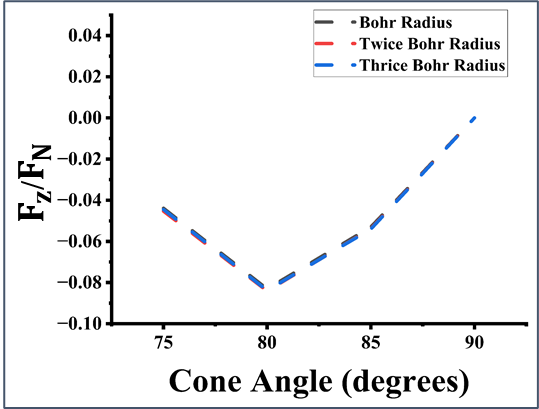


Figure 5S: Force for Coulomb Potential Energy of a decaying power of n, E/E0=12 (where E0 is the energy scale), R=Bohr Radius, and incident beam mass of 4 u. The force direction, as well as the magnitude, from this figure, is considerable as the negative $F_{z}$/FN in Fig. 3(b) of Ref. [1] ranges from 0 to 0.05. ${10}^{-3}$ or less is considered zero or negligible for this simulation. Further radial scaling(R=$2\times Bohr Radius$, and R=$3\times Bohr Radius$) verifies the independency of R.


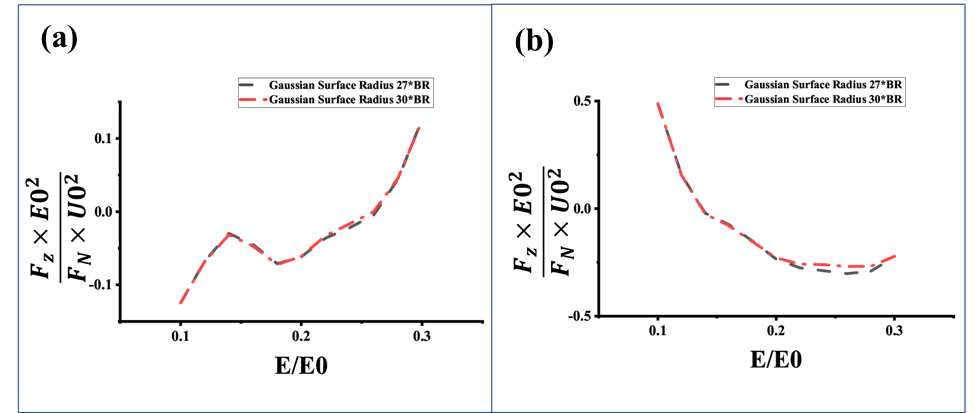


Figure 6S. Force for Morse Potential Energy with b/R= 2.2, R=Bohr Radius, incident beam mass=reduced mass of He and scatterer of 100 u system. The scale of our result is very similar(Negative $\frac{Fz*{E0}^{2}}{FN{*U0}^{2}}$ range is from 0 to 0.2 in [1]). However, for two different U0 (U0=0.4E0 in (a) and U0=0.8E0 in (b), which are chosen arbitrarily), the trend in the change in force direction and magnitude with respect to change in E/E0 are different. On the other hand, as expected, the force is independent of R as well as the Gaussian surface of a large radius ($27BR$ and $30BR$). Simulation for R=$2\times Bohr Radius$ gives the exact same results.

| **R parameter** | $\boldsymbol{F}_{\boldsymbol{x}}$ **(N)** | $\boldsymbol{F}_{\boldsymbol{y}}$ **(N)** | $\boldsymbol{F}_{\boldsymbol{z}}$ **(Pulling Force) (N)** |
| --- | --- | --- | --- |
| $Bohr Radius$ | -2.4989$\times{10}^{-24}$ | 3.3755$\times{10}^{-25}$ | -1.0941$\times{10}^{-22}$ |
| $2\times Bohr Radius$ | 3.4396$\times{10}^{-25}$ | 1.2883$\times{10}^{-24}$ | -1.1334$\times{10}^{-22}$ |
| $3\times Bohr Radius$ | 1.2468$\times{10}^{-24}$ | -8.2924$\times{10}^{-25}$ | -1.1368$\times{10}^{-22}$ |

Table 1S: Simulation result for the setup in I using Bessel Beam. The axial pulling force for R= Bohr Radius is consistent with the force magnitude obtained using two plane waves. Further scaling of R verifies the independence of radial scaling on the force.

## **SA3: 3D Simulation of our Set-up for Step Approximated He-Xe Potential Energy**

**Modeling Process:**

- *Space Dimension:*

In the Model Wizard Window, **3D** space dimension is selected.

- *Physics:*

**Schrodinger Equation (schr)** under **Semiconductor** is selected.

- Study:

Under General Studies, **Stationary Study** is selected,

- *Global Definitions*
- *Parameters 1*

A parameter .txt file is loaded, which contains the necessary information for the simulation, including Energy (E), Volumetric Density(nHE), and Mass (mHE) of the Incident Particles, *d*, and so on.

- *Component 1 (comp 1)*
- *Geometry*

Two Blocks for the modeling domain are created of the sizes described in Model Definition.

Two Spheres for the target atom and one Sphere for the Gaussian surface are created of the sizes described in Model Definition.

- *Definitions*

*Force Density Components derived from the QM Stress Tensor are loaded from a .txt file in Variables 1 under Definitions.*

- *Schrodinger Equation (schr)*

After going to the Settings Window of Schrodinger Equation (schr), under Model Properties, Electrons is selected as the Particle Type, E is inserted in Energy, and 0 is inserted in Charge Number. Under Domain selection, all domains are selected.

- Effective Mass 1

Isotropic is selected. **mHE_nr** is inserted in Electron effective mass.

- Potential Energy

Electron Potential Energy 1: User-defined is selected, and 0 is inserted.

Electron Potential Energy 2: User-defined is selected, and the Potential Energy Well value of He-Xe interaction (-$V_{0}$) is inserted. Under Domain selection, Manual is selected, and Sphere 2 is added.

Electron Potential Energy 3: User-defined is selected, and the Potential Energy formula of He-Cu interaction (generated in Wolfram Mathematica from the interpolation points taken from graph [3]) is inserted. Under Domain selection, Manual is selected, and the block on the right side, along with all the domains inside it, is added.

- Zero Probability 1

Under Boundary Selection, Manual is selected, and the boundary between the two blocks, and Sphere 1 are added. (***Note:*** *Zero Probability Boundary Condition satisfies zero transmission of the wave. This means that the probability current density is zero as well as there is no leakage of the wave function at the boundary. Thus the probability becomes instantly zero at the boundary, and no particle is absorbed.)*

- Open Boundary 1

Under Boundary Selection, Manual is selected, and the boundary from which the incident wave with k vector in the z direction will enter is added. Incoming wave is ticked. Plane wave is selected as the wave type. A0(given in the parameter file) is inserted in Incoming wave amplitude. 0, 0, and 1 for x, y, and z components, respectively, are inserted in Incoming wave direction. Outgoing is selected under Open Boundary Type.

- Open Boundary 2

Under Boundary Selection, Manual is selected, and the boundary from which the incident wave with k vector in the -z direction will enter is added. Incoming wave is ticked. Plane wave is selected as the wave type. A0(given in the parameter file) is inserted in Incoming wave amplitude. 0, 0, and -1 for x, y, and z components, respectively, are inserted in Incoming wave direction. Outgoing is selected under Open Boundary Type.

- Open Boundary 3

Under Boundary Selection, Manual is selected, and all the remaining outer boundaries are added. Plane wave is selected as the Wave type. Incoming wave remains unticked. Outgoing is selected under Open Boundary Type.

- Mesh 1

Under Sequence Type, Physics-controlled mesh is selected, Under Physics-controlled mesh, Extra fine is selected as the Element Size. *(Note: Extra to Extremely Fine can be selected for this set-up as the He-Xe potential energy is continuously space-varying. If more than one potential energy (both He-Cu and He-Xe potential energies) were continuously space-varying as in the simulation for Fig. 6(d) in the manuscript,* *Extremely fine mesh would be required.)*

- *Study 1*

Parametric Sweep: Under Parameter names d and E are selected. The parameter value list for *d* (in nm) is inserted as 0, 0.05, 0.1, 0.15, 0.2, 0.25 to 0.65, and 0.7 to 1 separately in seven different simulations due to constraints in the domain selection of potential energies. The parameter value list for E (in meV) is inserted as 0, 10, 25, 30, 40.

The Simulation is ready to compute. **Build Mesh** is clicked, and then **Compute** is used to run the stationary study.

- *Results*
- Derived Values

In the setting window of Surface Integration 1 under Derived Values, under Expressions, -0.5*real(fxsurf), -0.5*real(fysurf), and -0.5*real(fzsurf) are inserted which gives the time-averaged force components (fxsurf, fysurf, and fzsurf are the force density components given in the .txt file loaded in variables). Under Selection, Manual is selected, and Sphere 3 is added. To get the force components, **Evaluate** is clicked, which generates the table containing the results.

## **SA4: Verifying the Simulation Method Using Matter Wave Tractor Beam**

As the Space Dimension, Physics, Global Definitions, Component 1 (comp 1), Study, Mesh, and Results parts are similar to our set-up, the details are not mentioned in the following steps.

- *Schrodinger Equation (schr)*

After going to the Settings Window of Schrodinger Equation (schr), under Model Properties, Electrons is selected as the Particle Type, E is inserted in Energy, and 0 is inserted in Charge Number. Under Domain selection, all domains are selected.

- Effective Mass 1

Isotropic is selected. **mHE** is inserted in Electron effective mass. (*Note: mHE is the reduced mass*)

- Potential Energy

Electron Potential Energy 1: User-defined is selected, and 0 is inserted.

Electron Potential Energy 2: User-defined is selected, and the spherical potential energy of the scatterer (U0, given in the parameter list) is inserted. Under Domain selection, Manual is selected, and Sphere 4 is added.

- Open Boundary 1

Under Boundary Selection, Manual is selected, and the two boundaries from which the incident wave with k vector in the x and -z directions will enter are added. Incoming wave is ticked. Plane wave is selected as the wave type. A0(given in the parameter file) is inserted in Incoming wave amplitude. cos(theta), 0, and -sin(theta) for x, y, and z components, respectively, are inserted in Incoming wave direction (where theta is the cone angle). Outgoing is selected under Open Boundary Type.

- Open Boundary 2

Under Boundary Selection, Manual is selected, and the two boundaries from which the incident wave with k vector in the x and z directions will enter are added. Incoming wave is ticked. Plane wave is selected as the wave type. A0(given in the parameter file) is inserted in Incoming wave amplitude. cos(theta), 0, and sin(theta) for x, y, and z components, respectively, are inserted in Incoming wave direction. Outgoing is selected under Open Boundary Type.

- Open Boundary 3

Under Boundary Selection, Manual is selected, and all the remaining outer boundaries are added. Spherical wave is selected as Wave type with center (0,0,0). Incoming wave remains unticked. Outgoing is selected under Open Boundary Type.

- Mesh 1

Under Sequence Type, Physics-controlled mesh is selected, Under Physics-controlled mesh, Normal Fine is selected as the Element Size. *(Note: Normal to Extremely Fine can be selected for this set-up as the potential is not continuously space-varying.)*

- *Study 1*

Parametric Sweep: Under Parameter names, theta is selected. The parameter value list for theta (in degrees) is inserted as 0 to 90.

# **Section B**

The incoming plane waves can be written in terms of spherical waves using Rayleigh’s formula as [4].

$$\psi_{inc}\left( r,\theta\right)={Ae}^{ik1z}+Ae^{ik2z}$$

$=\sum_{l=o}^{\infty} {Ai}^{l}(2l+1)j_{l}(k1r)P_{l}(cos\theta)+\sum_{l=o}^{\infty} Ai^{l}(2l+1)j_{l}(k2r)P_{l}(cos\theta)$ (3)

where $A$ is the amplitude of the incident wave, $l$ is the orbital angular momentum quantum number, $j_{l}$ is the $l$-th order spherical Bessel function, and $P_{l}$ is the $l$-th order Legendre polynomial. For our model, $k2=-k1=k$. Since our incident wave function is independent of $\phi$ ($z=rcos\theta$), only the $m=0$term survives; therefore, we have omitted all the $m\neq0$ terms (Section 11.2, Ref. [5]).

$\psi_{inc}(r,\theta)=\sum_{l=o}^{\infty} {Ai}^{l}(2l+1)j_{l}(kr)P_{l}(cos\theta)+\sum_{l=o}^{\infty} Ai^{l}(2l+1)j_{l}(-kr)P_{l}(cos\theta)$ (4)

Expanding $j_{l}(kr)$ in terms of spherical Hankel’s functions

$$j_{l}(kr)=\frac{1}{2}[h_{l}^{(1)}(kr)+h_{l}^{(2)}(kr)] = \frac{1}{2kr}[{(-i)}^{l+1}e^{ikr}+i^{l+1}e^{-ikr}] for kr>>1$$

$$j_{l}(-kr)=\frac{1}{2}[h_{l}^{\left( 1 \right)}(-kr)+h_{l}^{\left( 2 \right)}(-kr\frac{1}{2}[({-1)}^{l}h_{l}^{\left( 2 \right)}(kr)+{({-1)}^{l}h}_{l}^{\left( 1 \right)}(kr)=\frac{({-1)}^{l}}{2kr}\left[ i^{l+1}e^{-ikr}+\left( -i \right)^{l+1}e^{ikr} \right] for kr>>1$$

For large kr, the asymptotic condition is applied where spherical Hankel’s function of the first and second kinds go to $e^{+ikr}/kr$ and $e^{-ikr}/kr$, respectively. Therefore, Eq. (4) becomes

$$\psi_{inc}\left( r,\theta\right)\approx\sum_{l=o}^{\infty} Ai^{l}(2l+1)\frac{1}{2kr}[\left( -i \right)^{l+1}e^{ikr}+i^{l+1}e^{-ikr}]P_{l}(cos\theta)+$$

$\sum_{l=o}^{\infty} Ai^{l}(2l+1)\frac{({-1)}^{l}}{2kr}[i^{l+1}e^{-ikr}+{(-i)}^{l+1}e^{ikr}]P_{l}(cos\theta)$ (5)

Simplifying

$\psi_{inc}(r,\theta)\approx\sum_{l=o}^{\infty} \begin{aligned} (\frac{A\left( 2l+1 \right)}{2ikr}[e^{ikr}-({-1)}^{l}e^{-ikr}]P_{l}(cos\theta)- \\ \frac{A\left( 2l+1 \right)}{2ikr}[({-1)}^{l}e^{ikr}{-e}^{-ikr}]P_{l}\left( cos\theta\right)) \end{aligned}$ (6)

There is no interaction between the incident particles as the incident beam is rarefied. In the presence of incident atom-target atom spherical potential energy, the outgoing spherical ($e^{ikr}$ terms) waves will pick up a phase shift. Since both the wave functions get scattered by the same spherical potential energy, therefore, the phase shifts are equal, which gives the total wave function:

$\psi\approx\sum_{l=o}^{\infty} \begin{aligned} (\frac{A\left( 2l+1 \right)}{2ikr}[e^{i\left( kr+2\delta_{l} \right)}-({-1)}^{l}e^{-ikr}]P_{l}(cos\theta)- \\ \frac{A\left( 2l+1 \right)}{2ikr}\left[ \left( {-1)}^{l}e^{i\left( kr+2\delta_{l} \right)}{-e}^{-ikr} \right]P_{l}\left( cos\theta\right) \right) \end{aligned}$(7)

We will now compare Eq. (6) and (7) with formulae of $\psi$in terms of $l$-th partial wave amplitude ($a_{l}$) (Section 11.2, Ref. [5]).

$$\psi\left( r,\theta\right)= A\left[ e^{ikz}+k\sum_{l=o}^{\infty} i^{l+1}(2l+1)a_{l}h_{l}^{\left( 1 \right)}(kr)P_{l}(cos\theta) \right]+$$

$A\left[ e^{-ikz}+k\sum_{l=o}^{\infty} i^{l+1}(2l+1)a_{2l}h_{l}^{\left( 1 \right)}(kr)P_{l}(cos\theta) \right]$ (8)

Using the asymptotic form of spherical Hankel’s function gives the following relation:

$a_{l}$=${{(-1)}^{l}a}_{2l}=\frac{1}{2ik}(e^{2i\delta_{l}}-1)$

Therefore, writing Eq. (8) as

$$\psi_{total wave function}(r,\theta)=A\{e^{ikz}+f_{1}(\theta)\frac{e^{ikr}}{r}\}+A\{e^{-ikz}+f_{2}(\theta)\frac{e^{ikr}}{r}\}$$

where,

$f_{1}(\theta)$=$\sum_{l=0}^{\infty} (2l+1)a_{l}P_{l}(cos\theta)$

$$f_{2}(\theta)=\sum_{l=0}^{\infty} {(-1)}^{l}(2l+1)a_{l}P_{l}(cos\theta)$$

Therefore, our total wave function becomes

$\psi(r,\theta)=A\{e^{ikz}+e^{-ikz}+\left[ \sum_{l=0}^{\infty} (2l+1)a_{l}P_{l}(cos\theta)+\sum_{l=0}^{\infty} (2l+1)a_{l}P_{l}(cos\theta) {(-1)}^{l} \right]\frac{e^{ikr}}{r}\}$ (9)

From Eq. (9), the overall scattering amplitude for the first scattering event can be written as:

$f_{overall}(\theta)=f_{1}(\theta)+f_{2}(\theta)$= $\sum_{l=0}^{\infty} (2l+1)a_{l}P_{l}(cos(\theta))[1+ {(-1)}^{l}]$ (10)

To get $f_{overall}(\theta)$ for our model, we need to find $a_{l}$ in terms of $j_{l}(kb)$, and $h_{l}^{(1)}(kb)$. The scatterer potential energy is modeled as follows:

$V(r) =\left\{ \begin{aligned} \infty, (r<b) \\ -V_{0} , \left( b\leq r\leq a \right) \\ 0 , (r>a) \end{aligned} \right.$

where $V_{0}$=2.458 meV, a=$9\times Bohr Radius$, and b=$6.697\times Bohr Radius$ m for our model.

Applying the following three appropriate boundary conditions at $r=a$ and $r=b$:

$\psi(r=a^{-})=\psi(r=a^{+})$ (i)

$\left. \frac{\delta\psi}{\delta r} \right|_{r=a^{-}}=\left. \frac{\delta\psi}{\delta r} \right|_{r=a^{+}}$ (ii)

$\psi\left( r=b \right)=0$ (iii)

$kr$ value for $r=a$ and $E=25 meV$is:

$$ka=(\sqrt{2mE}/\hbar)a$$

=($\sqrt{2\times4\times1.6605{\times10}^{-27}\times25{\times10}^{-3}\times1.6\times{10}^{-19}}$/1.0546×${10}^{-34}$)$\times9{\times5.2918 \times10}^{-11}$

$\approx33$ >> 1

Therefore, we can use the formulae (Eq. (10)) derived using the asymptotic form of spherical Hankel’s functions.

For the region ($r>a$) the solution of the radial wave function of Schrodinger Equation (Eq. (9)) should be as follows:

$$\psi\left( r,\theta\right)= A\left[ e^{ikz}+e^{-ikz}+k\sum_{l=o}^{\infty} i^{l+1}(2l+1)a_{l}h_{l}^{\left( 1 \right)}(kr)P_{l}(cos\theta)[1+ {(-1)}^{l}] \right]$$

For the region (a$\leq r\leq$ b), the solution of the radial wave function of Schrodinger Equation (Eq. (9)) should be as follows:

$$\psi\left( r,\theta\right)=A^{'}\left[ e^{ik^{'}z}+e^{-ik^{'}z}+k^{'}\sum_{l=o}^{\infty} i^{l+1}(2l+1)a_{l}^{'}h_{l}^{\left( 1 \right)}(k^{'}r)P_{l}(cos\theta)[1+ {(-1)}^{l}] \right]$$

Where $k^{'}$=$\sqrt{2m(E-(-V0))}$/$\hbar$

Now, the relationship between $a_{l}$ and ${a'}_{l}$will be determined using boundary conditions (i) and (ii). Boundary condition (iii) will provide the value of $a_{l}^{'}$.

**Applying Boundary Condition (i):**

$$A\left[ e^{ikz}+e^{-ikz}+k\sum_{l=o}^{\infty} i^{l+1}(2l+1)a_{l}h_{l}^{\left( 1 \right)}(kr)P_{l}(cos\theta)[1+ {(-1)}^{l}] \right]=$$

$$A^{'}\left[ e^{ik^{'}z}+e^{-ik^{'}z}+k^{'}\sum_{l=o}^{\infty} i^{l+1}(2l+1)a_{l}^{'}h_{l}^{\left( 1 \right)}(k^{'}r)P_{l}(cos\theta)[1+ {(-1)}^{l}] \right]$$

Writing $e^{ikz}$ and $e^{ik'z}$ in spherical coordinates, and simplifying:

$$A{\left( 2l+1 \right)P}_{l}\left( cos\theta\right)\left[ \sum_{l=o}^{\infty} i^{l}j_{l}\left( ka \right)[1+ \left( -1 \right)^{l}]+k\sum_{l=o}^{\infty} i^{l+1}a_{l}h_{l}^{\left( 1 \right)}\left( ka \right)[1+ \left( -1 \right)^{l}] \right]=$$

$$A^{'}{(2l+1)P}_{l}(cos\theta)\left[ \sum_{l=o}^{\infty} i^{l}j_{l}(k^{'}a)[1+ \left( -1 \right)^{l}]+k^{'}\sum_{l=o}^{\infty} i^{l+1}a_{l}^{'}h_{l}^{(1)}(k^{'}a)[1+ \left( -1 \right)^{l}] \right]$$

Multiplying by $P_{l'}(cos\theta)sin\theta d\theta$ and integrating from 0 to $\pi$ (Eq. (4.34), Ref. [5] which exploited the orthonormality condition for Legendre polynomial to collapse its sum).

$\int_{0}^{\pi} P_{l}(cos\theta)P_{l'}(cos\theta)sin\theta d\theta=(\frac{2}{2l+1})\delta_{ll'}$ (11)

Dropping l’ gives:

$$A\left[ i^{l}j_{l}\left( ka \right)[1+ \left( -1 \right)^{l}]+ki^{l+1}a_{l}h_{l}^{\left( 1 \right)}\left( ka \right)[1+ \left( -1 \right)^{l}] \right]=$$

$A^{'}\left[ i^{l}j_{l}\left( k^{'}a \right)[1+ \left( -1 \right)^{l}]+k^{'}i^{l+1}a_{l}^{'}h_{l}^{\left( 1 \right)}\left( k^{'}a \right)[1+ \left( -1 \right)^{l}] \right]$ (12)

**Applying Boundary Condition (ii):**

$\frac{\delta}{\delta r}\psi(r=a^{+})$ =$\frac{\delta}{\delta r}\psi(r=a^{-})$

$\frac{\boldsymbol{\delta}}{\boldsymbol{\delta r}}A{(2l+1)P}_{l}(cos\theta)\left[ \sum_{l=o}^{\infty} i^{l}j_{l}\left( ka \right)[1+ \left( -1 \right)^{l}]+k\sum_{l=o}^{\infty} i^{l+1}a_{l}h_{l}^{\left( 1 \right)}\left( ka \right)[1+ \left( -1 \right)^{l}] \right]$=

$$\frac{\delta}{\delta r}A^{'}{(2l+1)P}_{l}(cos\theta)\left[ \sum_{l=o}^{\infty} i^{l}j_{l}(k^{'}a)[1+ \left( -1 \right)^{l}]+k^{'}\sum_{l=o}^{\infty} i^{l+1}a_{l}^{'}h_{l}^{(1)}(k^{'}a)[1+ \left( -1 \right)^{l}] \right]$$

Applying Eq. (11) and dropping l’ gives

$J=K$ (13)

where,

$$J=A\frac{1}{2}k\left[ j_{l-1}\left( ka \right)-j_{l+1}(ka) \right]i^{l}[1+ \left( -1 \right)^{l}]+Aki^{l+1}a_{l}\frac{1}{2}k\left[ h_{l-1}^{\left( 1 \right)}\left( ka \right)-h_{l+1}^{\left( 1 \right)}(ka) \right][1+ \left( -1 \right)^{l}]$$

$$K={A^{'}i}^{l}\frac{1}{2}k^{'}\left[ j_{l-1}\left( k^{'}a \right)-j_{l+1}(k^{'}a) \right][1+ \left( -1 \right)^{l}]+{A^{'}k}^{'}i^{l+1}a_{l}^{'}\frac{1}{2}k^{'} \left[ h_{l-1}^{\left( 1 \right)}\left( k^{'}a \right)-h_{l+1}^{\left( 1 \right)}\left( k^{'}a \right) \right][1+ \left( -1 \right)^{l}]$$

Using Eq. (12) and (13), we get the following relation:

$P/Q=$ $R/S$ (14)

where,

$$P=i^{l}j_{l}\left( ka \right)[1+ \left( -1 \right)^{l}]+ki^{l+1}a_{l}h_{l}^{\left( 1 \right)}\left( ka \right)[1+ \left( -1 \right)^{l}]$$

$$R=i^{l}j_{l}\left( k^{'}a \right)[1+ \left( -1 \right)^{l}]+k^{'}i^{l+1}a_{l}^{'}h_{l}^{\left( 1 \right)}\left( k^{'}a \right)[1+ \left( -1 \right)^{l}]$$

$$Q=i^{l}\frac{1}{2}k\left[ j_{l-1}\left( ka \right)-j_{l+1}(ka) \right][1+ \left( -1 \right)^{l}]+ki^{l+1}a_{l}\frac{1}{2}k\left[ h_{l-1}^{\left( 1 \right)}\left( ka \right)-h_{l+1}^{\left( 1 \right)}(ka) \right][1+ \left( -1 \right)^{l}]$$

$$S=i^{l}\frac{1}{2}k^{'}\left[ j_{l-1}\left( k^{'}a \right)-j_{l+1}(k^{'}a) \right][1+ \left( -1 \right)^{l}]+k^{'}i^{l+1}a_{l}^{'}\frac{1}{2}k^{'} \left[ h_{l-1}^{\left( 1 \right)}\left( k^{'}a \right)-h_{l+1}^{\left( 1 \right)}\left( k^{'}a \right) \right][1+ \left( -1 \right)^{l}]$$

**Applying Boundary Condition (iii)**

$$A^{'}{(2l+1)P}_{l}(cos\theta)\left[ \sum_{l=o}^{\infty} i^{l}j_{l}\left( k^{'}b \right)[1+ \left( -1 \right)^{l}]+k^{'}\sum_{l=o}^{\infty} i^{l+1}a_{l}^{'}h_{l}^{\left( 1 \right)}\left( k^{'}b \right)[1+ \left( -1 \right)^{l}] \right]=0$$

Applying Eq. (11) and dropping $l^{'}$

$A^{'}\left[ i^{l}j_{l}\left( k^{'}b \right)[1+ \left( -1 \right)^{l}]+k^{'}i^{l+1}a_{l}^{'}h_{l}^{\left( 1 \right)}\left( k^{'}b \right)[1+ \left( -1 \right)^{l}] \right]=0$

${a'}_{l}=\frac{-[j_{l}\left( k^{'}b \right)[1+ \left( -1 \right)^{l}]]}{ik'h_{l}^{\left( 1 \right)}\left( k^{'}b \right)[1+ \left( -1 \right)^{l}]}$ (15)

We used Eq. (14), and (15) to get $a_{l}$, and finally $f_{overall}(\theta=\pi/2)$ in Wolfram Mathematica (Section SC1). For $E=25 meV$, the $l-$th terms are taken up to $ka$ $\approx$ 33 (where k=$\sqrt{2mE}$/$\hbar$). This is because it is a high-energy scattering for which $l\leq kr$ terms should be taken into account[6].

$f(\theta=\pi/2)=\sum_{l=0}^{33} (2l+1)a_{l}P_{l}(cos(\theta))[1+ {(-1)}^{l}]$ (16)

# **Section C**

### **SC1: Numerical Solution of the Scattering Amplitude using Wolfram Mathematica for the First Scenario**

| **E (meV)** | $\left\vert\boldsymbol{f(\theta=\pi/2)} \right\vert$ **(**$\sqrt{\boldsymbol{m}^{\boldsymbol{-3}}}$**)** | $\left\vert\frac{\boldsymbol{f(\theta=\pi/2)}}{\boldsymbol{a}} \right\vert$ **(**$\sqrt{\boldsymbol{m}^{\boldsymbol{-3}}}$**)** |
| --- | --- | --- |
| 25 | $4.2204\times{10}^{-10}$ | 0.8883 |

Table 2S: The table represents the analytical magnitudes of $f(\theta=\pi/2)$ and $\frac{f(\theta=\pi/2)}{a}$ for incident beam energy 25 meV.

### **SC2: 1D Reflection of the scattered wave from the First Event of the First Scenario**

- *Space Dimension:*

In the Model Wizard Window, **1D** space dimension is selected.

- *Physics:*

**Schrodinger Equation (schr)** under **Semiconductor** is selected.

- Study:

Under General Studies, **Stationary Study** is selected,

- *Global Definitions*
- *Parameters 1*

A parameter .txt file is loaded, which contains the necessary information for the simulation, including Energy (E), Volumetric Density(nHE), and Mass (mHE_nr) of the Incident Particles, *a* (distance of the Zero Probability Boundary from the metal surface), and so on.

- *Component 1 (comp 1)*
- *Geometry*

Interval 1: Under Interval, Coordinates is selected in Specify, and Table is selected in Coordinates source. 0, a, and 0.9 are inserted in the first three rows.

- *Schrodinger Equation (schr)*

After going to the Settings Window of Schrodinger Equation (schr), under Model Properties, Electrons is selected as the Particle Type, E is inserted in Energy, and 0 is inserted in Charge Number. Under Domain selection, all domains are selected.

- Effective Mass 1

Isotropic is selected. **mHE_nr** is inserted in Electron effective mass.

- Potential Energy

Electron Potential Energy 1: User-defined is selected, and 0 is inserted.

Electron Potential Energy 2: User-defined is selected, and the Potential Energy formula of He-Cu interaction is inserted. Under Domain selection, Manual is selected, and the line on the right side is added.

- Zero Probability 1

Under Boundary Selection, Manual is selected, and the point between the two lines is added.

- Open Boundary 1

Under Boundary Selection, Manual is selected, and the point boundary from which the incident wave with k vector in the -x direction will enter is added. Incoming wave is ticked. Plane wave is selected as the wave type. A0*0.8883/(1-x) is inserted in Incoming wave amplitude (the origin of this simulation is at the surface, which is the reason for division by 1-x). -1 is inserted in Incoming wave direction. Outgoing is selected under Open Boundary Type. (***Note:*** 0.8883 is $\frac{f(\theta=\pi/2)}{a}$ for E=25meV).

- Open Boundary 3

Under Boundary Selection, Manual is selected, and the remaining outer point boundary is added. Incoming wave remains unticked. Outgoing is selected under Open Boundary Type.

- Mesh 1

Under Sequence Type, Physics-controlled mesh is selected, Under Physics-controlled mesh, Extremely fine is selected as the Element Size.

The Simulation is ready to compute. **Build Mesh** is clicked, and then **Compute** is used to run the stationary study.

- *Results*
- Derived Values

In the setting window of Global Evaluation under Derived Values, under Expressions, abs(schr.open1.int(psi/schr.psiI)-1)^2 is inserted, which gives the reflection coefficient. **Evaluate** is clicked, which generates the Reflection Coefficient for$E=25 meV$ in Table 3S.

### **SC3: Analysis of the Numerical Data for the First Scenario**

| **E(meV)** | **Reflection Coefficient** |
| --- | --- |
| 25 | 0.99927 |

Table 3S: The table represents the reflection coefficient (i.e., $R_{c}$) for incident beam energy 25 meV.

| **E (meV)** | $\boldsymbol{F}_{\boldsymbol{z}}$ **(N)** |
| --- | --- |
| 25 | $1.89\times{10}^{-19}$ |

Table 4S: The table represents the pushing force ($F_{z}$) experienced by the scatterer upon incidence of a single beam of energy 25 meV and no presence of Cu surface.

Using the data from Table 2S, 3S, and 4S, the analytical magnitude of ${(R_{c}\times\left| \frac{f(\theta=\pi/2)}{2\times d-a}e^{i\delta} \right|)}^{2}\times Fz$ for E = 25 meV and *d* = 10 Å is $1.46\times{10}^{-20}$ N, which is around $1.3$ times the COMSOL simulation result. The difference can be explained by the approximations made regarding the reflection of scattered waves. Tractor beam

### **SC4: Formulae of He-Cu Potential Energy and He-Xe Potential Energy obtained from Wolfram Mathematica**

The purpose of using formulae instead of interpolation from graphs is the constant extrapolation constraint. This introduces errors where the graph goes to infinity. Therefore, we modeled potential energy using formulae, which are obtained in the form of (12,6) Lennard Jones Potential Energy using Wolfram Mathematica. **NonlinearModelFit** has been applied to the data points collected from He-Cu and He-Xe potential energy graphs. The formulae are given below:

$U(r)=\frac{u}{r^{12}}$-$\frac{v}{r^{6}}$ for He-Xe

$U(x+d)=\frac{e}{{(x+d)}^{12}}$-$\frac{f}{{(x+d)}^{6}}$ for He-Cu

where d is the distance between the copper surface and the scatterer center, and u, v, e, and f are parameters that depend on the potential energy curve. $U(r)$ and $r$ are in atomic units. $U(x+d)$ and $x$ are in meV and m, respectively.

u =2.94085$\times{10}^{10}$

v =325882

e =$1.22319\times{10}^{-114}$

f = $4.21315\times{10}^{-57}$

# **References**

[1] Gorlach, A. A., Gorlach, M. A., Lavrinenko, A. V., & Novitsky, A. (2017). Matter-wave tractor beams. *Physical Review Letters*, *118*(18), 180401. DOI: 10.1103/PhysRevLett.118.180401

[2] Double Barrier 1D. (n.d.). COMSOL. Retrieved April 1, 2023, from <https://www.comsol.com/model/double-barrier-1d-47041>

[3] Zaremba, E., & Kohn, W. (1977). Theory of helium adsorption on simple and noble-metal surfaces. *Physical Review B*, *15*(4), 1769. DOI: 10.1103/PhysRevB.15.1769

[4] Arfken, G. B., & Weber, H. (2005). Mathematical methods for physicists (6th ed.). Academic Press.

[5] Griffiths, D. J. (2005). *Introduction to Quantum Mechanics* (2nd ed.). Pearson.

[6] Fitzpatrick, R. (n.d.). Hard sphere scattering. Retrieved April 30, 2023, from <https://farside.ph.utexas.edu/teaching/qmech/Quantum/node136.html>
